# Estimated Reductions in Opioid Overdose Deaths With Sustainment of Public Health Interventions in 4 US States

**DOI:** 10.1001/jamanetworkopen.2023.14925

**Published:** 2023-06-09

**Authors:** Jagpreet Chhatwal, Peter P. Mueller, Qiushi Chen, Neeti Kulkarni, Madeline Adee, Gary Zarkin, Marc R. LaRochelle, Amy B. Knudsen, Carolina Barbosa

**Affiliations:** 1 Institute for Technology Assessment, Massachusetts General Hospital, Boston; 2 Harvard Medical School, Boston, Massachusetts; 3 Harold and Inge Marcus Department of Industrial and Manufacturing Engineering, The Pennsylvania State University, University Park; 4 RTI International, Research Triangle Park, North Carolina; 5 Clinical Addiction Research and Education Unit, Section of General Internal Medicine, Department of Medicine, Boston University School of Medicine, Boston, Massachusetts; 6 Boston Medical Center, Boston, Massachusetts

## Abstract

**Importance:**

In 2021, more than 80 000 US residents died from an opioid overdose. Public health intervention initiatives, such as the Helping to End Addiction Long-term (HEALing) Communities Study (HCS), are being launched with the goal of reducing opioid-related overdose deaths (OODs).

**Objective:**

To estimate the change in the projected number of OODs under different scenarios of the duration of sustainment of interventions, compared with the status quo.

**Design, Setting, and Participants:**

This decision analytical model simulated the opioid epidemic in the 4 states participating in the HCS (ie, Kentucky, Massachusetts, New York, and Ohio) from 2020 to 2026. Participants were a simulated population transitioning from opioid misuse to opioid use disorder (OUD), overdose, treatment, and relapse. The model was calibrated using 2015 to 2020 data from the National Survey on Drug Use and Health, the US Centers for Disease Control and Prevention, and other sources for each state. The model accounts for reduced initiation of medications for OUD (MOUDs) and increased OODs during the COVID-19 pandemic.

**Exposure:**

Increasing MOUD initiation by 2- or 5-fold, improving MOUD retention to the rates achieved in clinical trial settings, increasing naloxone distribution efforts, and furthering safe opioid prescribing. An initial 2-year duration of interventions was simulated, with potential sustainment for up to 3 additional years.

**Main Outcomes and Measures:**

Projected reduction in number of OODs under different combinations and durations of sustainment of interventions.

**Results:**

Compared with the status quo, the estimated annual reduction in OODs at the end of the second year of interventions was 13% to 17% in Kentucky, 17% to 27% in Massachusetts, 15% to 22% in New York, and 15% to 22% in Ohio. Sustaining all interventions for an additional 3 years was estimated to reduce the annual number of OODs at the end of the fifth year by 18% to 27% in Kentucky, 28% to 46% in Massachusetts, 22% to 34% in New York, and 25% to 41% in Ohio. The longer the interventions were sustained, the better the outcomes; however, these positive gains would be washed out if interventions were not sustained.

**Conclusions and Relevance:**

In this decision analytical model study of the opioid epidemic in 4 US states, sustained implementation of interventions, including increased delivery of MOUDs and naloxone supply, was found to be needed to reduce OODs and prevent deaths from increasing again.

## Key Points

**Question:**

What is the association of sustaining public health interventions with the reduction in opioid-related overdose deaths (OODs) in 4 US states?

**Findings:**

In this decision analytical model that simulated the opioid epidemic in Kentucky, Massachusetts, New York, and Ohio, 4 states highly affected by the opioid epidemic, a 2- to 5-fold increase in initiation and retention of medications for opioid use disorder along with increased supply of naloxone could reduce OODs by an estimated 13% to 17% in Kentucky, 17% to 27% in Massachusetts, 15% to 22% in New York, and 15% to 22% in Ohio after 2 years, compared with the status quo. Sustaining these interventions for 3 additional years could reduce the annual number of OODs at the end of 5 years by 18% to 27% in Kentucky, 28% to 46% in Massachusetts, 22% to 34% in New York, and 25% to 41% in Ohio.

**Meaning:**

These findings suggest that sustained implementation of a combination of interventions is critical for achieving a reduction in the annual number of opioid overdose deaths and preventing deaths from increasing again in states highly affected by the opioid epidemic.

## Teaser

This decision analytical model estimates the change in the projected number of opioid-related overdose deaths under different durations of public health interventions, including increasing initiation and retention of medications for opioid use disorder and naloxone distribution efforts.

## Introduction

In 2017, the opioid epidemic was declared a US public health emergency.^1^ Despite ongoing efforts, opioid overdose deaths continue to rise. The increased social isolation and limited access to substance abuse treatment resulting from the COVID-19 pandemic, and increased penetration of fentanyl, have made the situation even more dire.^2^ In 2021, more than 80 000 people were estimated to have died from an opioid-related overdose, the highest number recorded in a 12-month period and an increase of nearly 15% compared with the previous year.^3^

To address the ongoing crisis, a multipronged approach consisting of evidence-based practices (EBPs) across prevention, harm reduction, and treatment is needed.^4^ Prevention initiatives, such as prescription drug monitoring programs and improved professional guidelines on opioid prescribing, seek to limit the number of people using opioids inappropriately.^5,6,7^ Harm reduction strategies that include overdose prevention with naloxone can reduce opioid overdose fatalities.^8,9,10^ Treatment includes effective medications for opioid use disorder (MOUDs), primarily buprenorphine and methadone, which can decrease all-cause and overdose mortality for persons with opioid use disorder (OUD).^11^ However, in 2020, only approximately 11% of the 2.7 million people aged 12 years or older with OUD received MOUDs.^12^ For those who did receive MOUD treatment, low retention rates undermine treatment effectiveness,^11,13^ especially given the increased risk of an overdose immediately after MOUD discontinuation.^11,14^

To reduce opioid overdose deaths, several efforts have been launched by the National Institutes of Health Helping to End Addiction Long-term (HEAL) Initiative. In 2019, one of the most ambitious implementation studies, the HEALing Communities Study^15^—a multisite, parallel-group, cluster randomized waiting-list trial—was launched in 67 communities (eg, counties and towns) across 4 US states: Kentucky, Massachusetts, New York, and Ohio.^16^ The primary goal of the HEALing Communities Study is to assess the feasibility of reducing the number of OODs by supporting communities to select and implement 3 EBPs^16^: (1) overdose education and naloxone distribution (OEND); (2) effective delivery of MOUDs; and (3) enhanced opioid prescription safety.

While wide implementation of multiple evidence-based interventions is needed to reduce OODs,^17,18,19,20^ most studies support the implementation of interventions only for a limited period of time.^15,21^ For example, the HEALing Communities Study supports the implementation of interventions for approximately 2 years. Some communities may opt to sustain interventions after the study, but no additional funding will be provided as part of the HEALing Communities Study. Because sustaining interventions requires substantial resources, understanding the effect of sustaining—or not sustaining—interventions on future OODs is important for policy making. In addition, as resources are limited, knowing which interventions are more effective and, if being sustained, can result in greater impact than others may inform the prioritization of EBPs selected for sustainment. Therefore, our objective was to evaluate the effectiveness of widely implementing and sustaining different EBPs at the state level on reducing OODs. We focused our analysis on the 4 states with communities participating in the HEALing Communities Study.

## Methods

### Model Overview

We developed the Opioid Policy Model (OPyM), a system dynamics mathematical model, that simulates the opioid epidemic in 4 states: Kentucky, Massachusetts, New York, and Ohio. OPyM simulates historical trends of OODs in each state from 2015 to 2020, accounting for reduced initiation of MOUDs and the increase in OODs associated with the COVID-19 outbreak starting in 2020.^22,23^ Motivated by the HEALing Communities Study, we used the model to project the number of OODs under different levels of EBPs for an initial intervention period of 2 years (August 2020 to June 2022) and a sustainment period of up to 3 years beyond the initial period. Modeled interventions included increasing initiation of and retention on MOUD, increasing distribution of naloxone overdose rescue kits, and preventing prescription opioid misuse. By simulating different combinations of interventions, we estimated the reduction in OODs after the initial 2-year period and with a sustainment duration for 0 to 3 additional years, compared with the status quo. This study protocol was approved by Advarra, Inc, the HEALing Communities Study’s single institutional review board. We followed the Consolidated Health Economic Evaluation Reporting Standards (CHEERS) reporting guideline for reporting model design and analysis results.^24^

### OPyM

**Figure 1.**
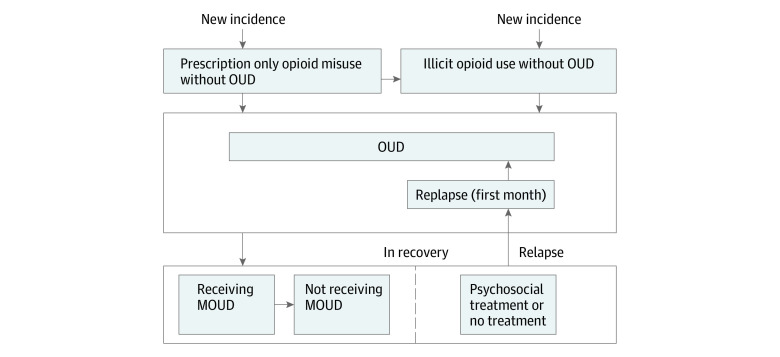
Opioid Policy Model Schematic Showing Transition Between Different Health States The state-level Opioid Policy Model consists of 4 categories of health states representing subpopulations at different stages of opioid use: (1) prescription opioid misuse, (2) illicit opioid use, (3) opioid use disorder (OUD), and (4) in recovery. Individuals can enter the model over time with either prescription opioid misuse or illicit opioid use and may subsequently develop OUD. Those with OUD can transition to the in-recovery state, starting the recovery process typically through treatment initiation. Individuals in recovery remain at risk of relapse, returning to the OUD state. We assume that those who are no longer receiving medications for opioid use disorder (MOUD) must relapse before receiving MOUDs again. All individuals in the model who are actively using opioids have a risk of opioid overdose death that depends on their health state, and all individuals have a background mortality risk from other (ie, nonopioid-related) causes (transition not shown in figure for simplicity). Deaths (from opioid overdose or from other causes) are possible from all states.

We expanded a previously developed population-level system dynamics mathematical model of the US opioid epidemic^25^ by incorporating (1) state-level data to simulate the epidemic in 4 states and (2) EBP interventions as defined in the HEALing Communities Study. The state-level OPyM consists of 4 categories of health states representing subpopulations at different stages of opioid use (Figure 1; eFigure 1 in Supplement 1): (1) prescription opioid misuse, (2) illicit opioid use, (3) OUD, and (4) in recovery. New individuals can enter the model over time with either prescription opioid misuse or illicit opioid use and may subsequently develop OUD. Those with OUD can transition to the in-recovery state, starting the recovery process typically through treatment initiation. Individuals in recovery remain at risk of relapse, returning to the OUD state. We assume that those who are in recovery and no longer taking MOUDs must relapse before going back to receiving MOUDs. During the first month of relapse, individuals have a higher risk of OOD compared with subsequent months.^26^ All individuals in the model who are actively using opioids have a risk of OOD that depends on their health state, and all individuals have a background mortality risk from other (ie, nonopioid-related) causes. Transitions between health states are simulated with a monthly cycle.

### Treatment and Recovery

Each month, individuals with OUD may initiate treatment and enter either the MOUD or the non-MOUD treatment states (Figure 1). The MOUD states include the 2 most common treatment types: methadone and buprenorphine. Because individuals receiving MOUDs may discontinue treatment, and the risk of relapse to OUD and overdose varies with treatment duration,^26,27,28^ we further differentiated the MOUD health states by treatment duration (from 1 to 6 months by each month and longer than 6 months) (eAppendix 1 in Supplement 1), and included an off-MOUD compartment for individuals who discontinue MOUD treatment. The rates of MOUD discontinuation and relapse were estimated from published studies for each treatment type^27,28,29^ and were aggregated with weights based on treatment volumes at the state level that were estimated from the National Survey of Substance Abuse Treatment Services (N-SSATS)^30^ for methadone and Automated Reports and Consolidated Ordering System (ARCOS) Retail Drug Summary Reports^31^ for buprenorphine (eFigure 2 in Supplement 1). We included a non-MOUD treatment state to represent individuals’ recovery process through treatment and services other than medication, such as detoxification, residential programs, and psychosocial treatment.^32^ The monthly rates of recovery following non-MOUD treatment (eg, psychosocial, peer supports) and no treatment we derived from analysis of the first and second waves of the National Epidemiologic Survey on Alcohol and Related Conditions.^33^

### Baseline Population in Each Health State

The initial size of the OUD population in Massachusetts in 2015 was based on published estimates of OUD prevalence from a capture-recapture study.^34^ Such state-level prevalence estimates were not available for all states. Therefore, we relied on the estimates of state-level OUD prevalence from the National Survey on Drug Use and Health (NSDUH) (J. Villani, PhD, National Institute on Drug Abuse, email, March 13, 2020).^35^ Because prevalence estimates from NSDUH are likely to be underestimated due to the self-report outcomes and the survey in only the noninstitutionalized civilian population,^34,36^ we adjusted each state-specific NSDUH prevalence estimate by a multiplier of 5.42,^36^ where the multiplier was estimated by comparing Massachusetts OUD prevalence estimate from the capture-recapture study with the OUD prevalence estimate of Massachusetts reported from NSDUH (J. Villani, PhD, National Institute on Drug Abuse, email, March 13, 2020).^35^ Following a similar multiplier approach, we adjusted the NSDUH-estimated prevalence of prescription opioid misuse and of illicit opioid use (without OUD) (eAppendix 2 in Supplement 1).

### Opioid Overdose Mortality

We estimated the baseline risk of OOD for each health state by calibrating the model to reproduce the observed annual number of OODs in the period from January 2015 to December 2020. To account for increasing use of illicit opioids, including fentanyl, we increased the overdose mortality rate over time, representing increasing lethality of opioid misuse. The opioid overdose mortality risk in the model was adjusted based on MOUD treatment status (eg, on-MOUD vs off-MOUD and duration of treatment) using relative risks that were estimated from a meta-analysis of treatment studies.^26^ In addition, considering the increasing trend of OODs since the COVID-19 pandemic outbreak,^2^ we gradually increased the baseline overdose mortality rate from January to March 2020 and assumed it remained at the elevated rate until June 2026 based on recent trends.^37,38,39,40,41^ The magnitude of the increase was estimated by calibrating the model to the observed number of overdose deaths in the year 2020.

### Model Calibration

For parameters that could not be estimated directly from clinical studies or empirical data, we inferred their values through calibration to observable data. Calibrated parameters included the incidence of illicit opioid use, transition rates between opioid use health states, the MOUD treatment initiation rate, and the baseline and growth rate of overdose mortality rates (eAppendix 2 and eTable 4 in Supplement 1). To account for the COVID-19 pandemic, we assumed that treatment initiation rates were reduced by 28% in early 2020 and returned to approximately 90% of prepandemic rates by June 2020 (eAppendix 2 in Supplement 1).^22,23^ Calibration was performed separately for each of the 4 US states. Calibration targets included multiple observed outcomes between January 2015 and December 2020, including (1) the number of overdose deaths from any opioid and from illicit opioids that were extracted from the CDC Wide-Ranging Online Data for Epidemiologic Research (WONDER) Multiple Cause of Deaths Database^42^; (2) prevalence of prescription opioid misuse, illicit opioid use (without OUD), and OUD; and (3) number of people receiving MOUDs as estimated from ARCOS and N-SSATS data.^31,43^ We applied a directed search algorithm^44^ to determine the best sets of parameter values such that the simulated model outcomes matched the calibration target values as closely as possible (eFigure 3 and eTables 1-3 in Supplement 1). To account for the uncertainty in the parameter calibration, we replicated the calibration process 1250 times and selected the best 1000 sets of calibrated parameters for model evaluation. We present mean values of the 1000 runs for all key model outcomes.

### Simulated Intervention Scenarios

We simulated the following interventions individually as well as combined in an additive order in the initial intervention period from August 2020 to June 2022 aligning with the timeline of HEALing Communities Study (eAppendix 3 and eFigure 5 in Supplement 1): (1) increased treatment recovery support that results in a MOUD treatment retention rate at the level observed in clinical trials^29^; (2) expanded treatment outreach efforts that yield a 2-fold increase in MOUD initiation rates compared with the baseline rate in each state; (3) an increase in OEND that results in a 10% reduction in the overdose mortality rate; and (4) prescription opioid safety efforts that reduce the incidence of prescription opioid misuse by 50%.^25^ An aspirational 5-fold increase in MOUD initiation rates was also tested to explore the potential impact of a more aggressive intervention to increase MOUD use.

We further examined scenarios in which interventions are sustained for up to 3 years beyond the end of the initial intervention period in June 2022. Specifically, we assessed the strategies of (1) sustaining prevention efforts only, (2) sustaining increased naloxone distribution efforts only, (3) sustaining both the increased MOUD treatment initiation rate (by either 2- or 5-fold) and the increased retention rate, and (4) sustaining all interventions for an additional 1, 2, or 3 years.

### Statistical Analysis

The model was programmed in R version 4.1.1 (R Foundation for Statistical Computing) using deSolve package for numerically solving the system dynamics model. The primary model outcome was the projected number of OODs in each month until June 2026. In comparison with the status quo (current practice) in each state, we evaluated the reduction in OODs achieved under each intervention scenario at the end of the 2-year intervention period. We further compared the reduction in OODs if interventions were sustained for 1, 2, or 3 years.

## Results

Model-projected OODs closely replicated the outcomes reported by the CDC from 2015 to 2020 for each state (eFigure 4 in Supplement 1). An increase in OODs was observed during 2020 because of COVID-19. The model also closely replicated observed temporal trends of other calibration targets, including the number of people receiving MOUDs and the prevalence of OUD (eFigure 4 in Supplement 1). We estimated that in 2020, 3.4% of people with OUD were receiving MOUDs in Kentucky, 6.8% in Massachusetts, 8.6% in New York, and 3.6% in Ohio.

**Figure 2.**
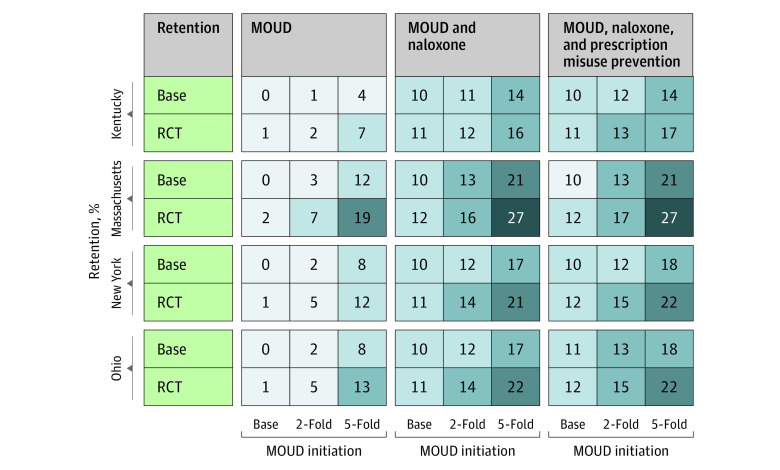
Estimated Percentage Reduction in Annual Opioid Overdose Deaths After 2 Years of Implementation of Evidence-Based Practice Interventions in Kentucky, Massachusetts, New York, and Ohio Compared With the Status Quo For each state and the intervention combinations, columns represent the scenarios of increasing medications for opioid use disorder (MOUD) initiation (a plausible 2-fold increase and an aspirational 5-fold increase) and rows represent the 6-month retention of MOUDs (base case retention of 32% for buprenorphine and 52% for methadone; and high retention observed in randomized clinical trials [RCTs] at 46% for buprenorphine and 74% for methadone). The first set of columns shows the estimated percentage reduction of implementing of MOUD-related interventions. The second set of columns add overdose education and naloxone distribution, which translates to a 10% mortality rate reduction. The third set of columns adds an increase in safe opioid prescribing that translates to a 50% reduction in new prescription opioid misuse.

Figure 2 shows the estimated reduction in annual OODs after 2 years of implementation of 3 EBPs—effective delivery of MOUD, OEND, and prescription opioid safety—compared with the status quo of no implementation of HEALing Communities Study interventions in each of the 4 states. A 2-fold increase in MOUD initiation rates, along with an increase in the MOUD treatment retention rate to the level observed in clinical trials, would marginally reduce annual OODs by 2% to 7% across the 4 states. A 5-fold aspirational increase in MOUD initiation rates could decrease annual OODs by 7% to 19% at the end of this 2-year period. Consistent with our assumption, the addition of OEND to MOUD delivery could further reduce overdose deaths by approximately 10%, but the prescription opioid safety program was associated with only marginal reductions in OODs in each state (≤1% reduction). The reduction in annual OODs after 2 years of the implementation of all EBPs, including the 5-fold increase in MOUD initiation, was estimated to be 17% in Kentucky, 27% in Massachusetts, 22% in New York, and 22% in Ohio.

**Figure 3.**
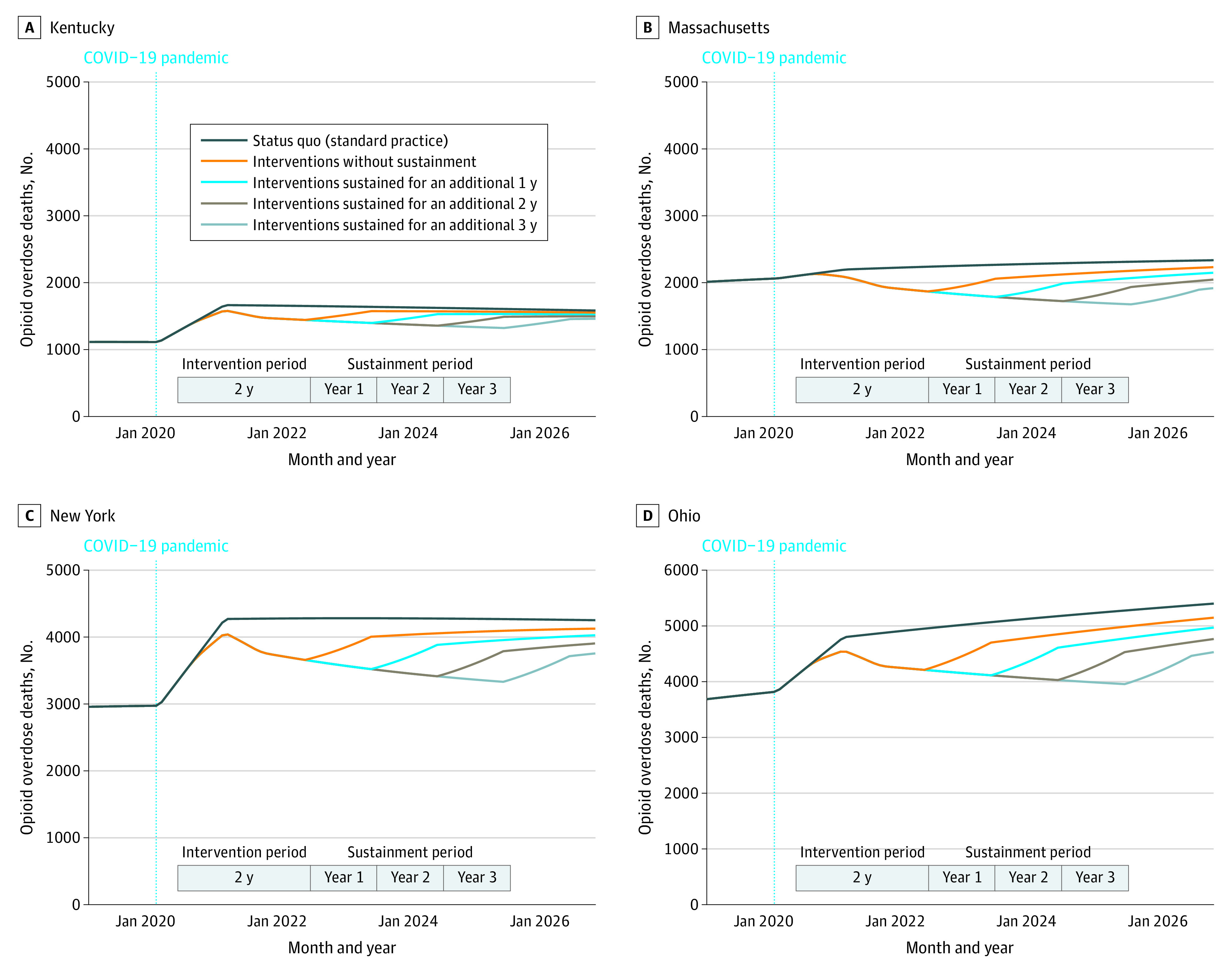
Temporal Trends in Estimated Opioid Overdose Deaths Under the Status Quo and With the Implementation of Interventions, With and Without Sustainment for Different Durations The selected intervention consists of 2-fold increase in medications for opioid use disorder, medications for opioid use disorder retention at the level observed in clinical trials (6-month retention of 46% for buprenorphine and 74% for methadone), overdose education and naloxone distribution that translate to a 10% mortality rate reduction, and increase in safe opioid prescribing that translates to a 50% reduction in new prescription opioid misuse.

Figure 3 shows the model-estimated temporal trends in OODs in each state under the status quo and with the implementation of EBPs sustained for alternative durations (1-3 years) beyond the initial 2-year intervention period. Under the status quo, the annual number of OODs remained relatively unchanged in Kentucky, Massachusetts, and New York but increased substantially in Ohio. The longer the EBP interventions were sustained, the more OODs were estimated to be prevented. As expected, compared with a 2-fold increase, a 5-fold increase in MOUD initiation would be associated with a more pronounced reduction in annual OODs (eFigure 6 in Supplement 1). However, in both cases, OODs were estimated to increase after termination of the interventions.

**Figure 4.**
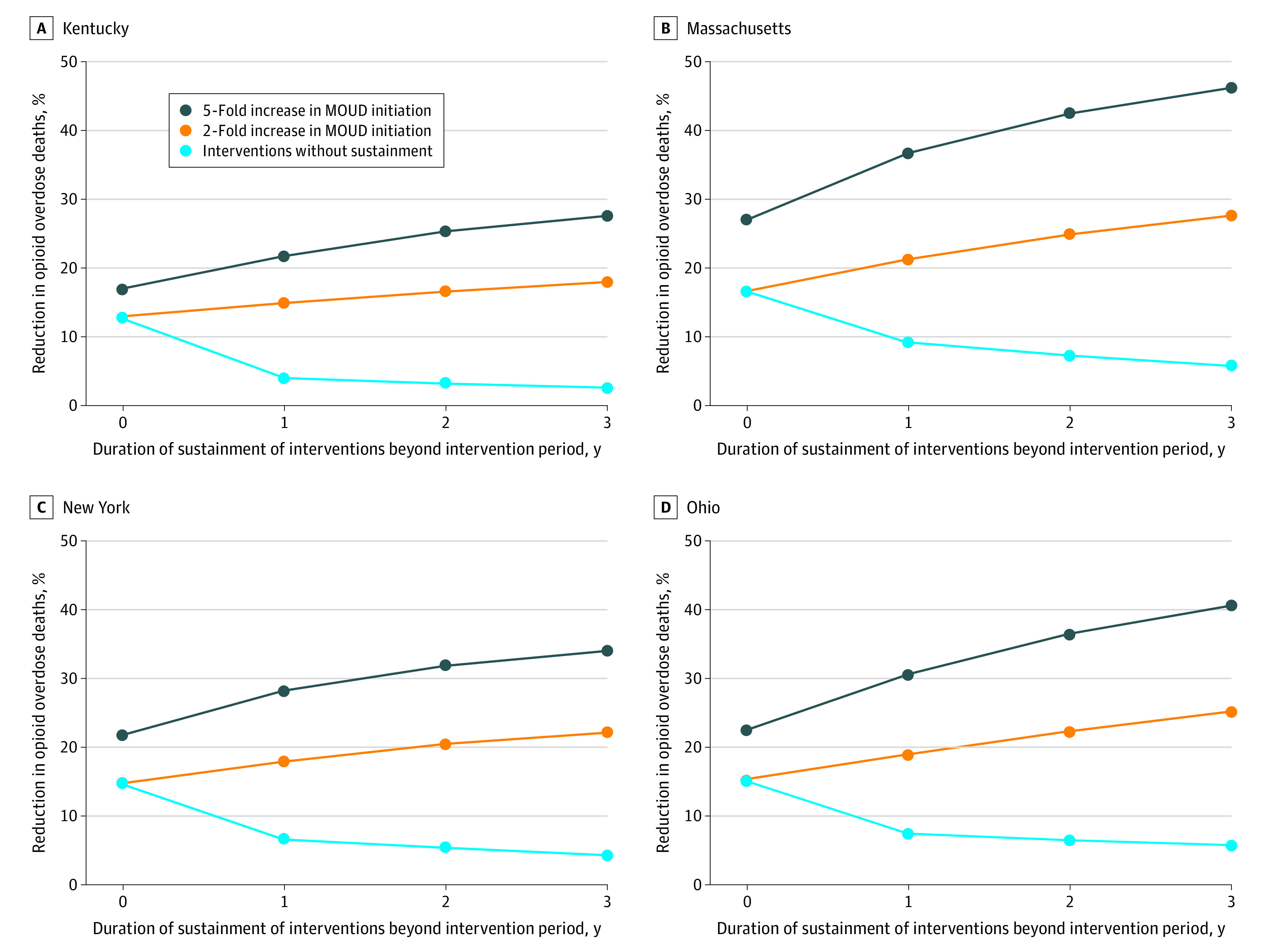
Estimated Reduction in Annual Opioid Overdose Deaths in Each State, With and Without Sustainment of the Intervention Relative to the Status Quo The selected intervention consists of 2-fold increase in medications for opioid use disorder (MOUD) initiation rates, MOUD retention at the level observed in clinical trials (6-month retention of 46% for buprenorphine and 74% for methadone), overdose education and naloxone distribution that translate to a 10% mortality rate reduction, and an increase in safe opioid prescribing that translates to a 50% reduction in new prescription opioid misuse.

Figure 4 summarizes the expected reduction in annual OODs under alternative durations of sustainment of evidence-based interventions after the 2-year initial intervention period. When MOUD initiation is increased by 2-fold, sustaining all other interventions for 1 to 3 years was estimated to reduce annual OODs (compared with the status quo) by 15% to 18% in Kentucky, 21% to 28% in Massachusetts, 18% to 22% in New York, and 19% to 25% in Ohio. Similarly, if MOUD initiation is increased by 5-fold, sustaining all other interventions for 1 to 3 years was estimated to reduce annual OODs (compared with the status quo) by 22% to 27% in Kentucky, 37% to 46% in Massachusetts, 28% to 34% in New York, and 31% to 41% in Ohio. In all states, not sustaining interventions could diminish the positive outcomes associated with interventions by bringing the annual OOD rates close to that projected under the status quo, resulting in a marginal 3% to 6% reduction in annual OODs across the 4 states.

In addition to reductions in the annual number of OODs relative to the status quo, we also estimated the cumulative number of OODs averted by the implementation of different interventions (individually or in combination) for various durations. Compared with the status quo, implementing EBPs without sustainment of interventions, with the increase in MOUD initiation ranging from 2- to 5-fold, could avert 455 to 746 deaths in Kentucky, 1056 to 2051 deaths in Massachusetts, 1608 to 2886 deaths in New York, and 2084 to 3724 deaths in Ohio by June 2025 (Table). Sustaining all interventions for 3 years beyond the initial intervention period could avert 1049 to 1499 deaths in Kentucky, 2231 to 3689 deaths in Massachusetts, 3490 to 5280 deaths in New York, and 4158 to 7132 deaths in Ohio by June 2025. If only 1 intervention is sustained beyond the initial intervention period, then maintaining the increased supply of naloxone kits was estimated to have the largest effect on OODs in most simulated scenarios. In contrast, sustaining prescription opioid safety was estimated to have the smallest effect.

**Table.** Estimated Opioid Overdose Deaths Averted Between August 2020 and June 2025 by the Duration of Sustaining Different Interventions in Kentucky, Massachusetts, New York, and Ohio

Sustained interventions	Deaths averted between August 2020 and June 2025 under different durations of sustainment of interventions							
	2-fold MOUD initiation increase				5-fold MOUD initiation increase			
	Intervention period only	Sustainment, y			Intervention period only	Sustainment, y		
		1	2	3		1	2	3
**Kentucky**								
Sustain all 4 interventions	455	691	892	1049	746	1084	1336	1499
Increased MOUD initiation		486	506	511		850	913	932
Increased MOUD retention^a^		485	504	511		793	817	825
OEND^b^		600	746	892		882	1021	1162
Safe opioid prescribing^c^		474	486	490		765	777	781
**Massachusetts**								
Sustain all 4 interventions	1056	1572	1972	2231	2051	2867	3414	3689
Increased MOUD initiation		1185	1270	1299		2418	2656	2736
Increased MOUD retention^a^		1172	1247	1276		2213	2301	2334
OEND^b^		1258	1468	1685		2224	2411	2610
Safe opioid prescribing^c^		1091	1108	1112		2087	2104	2108
**New York**								
Sustain all 4 interventions	1608	2401	3035	3490	2886	4026	4817	5280
Increased MOUD initiation		1764	1862	1893		3319	3585	3667
Increased MOUD retention^a^		1757	1850	1885		3092	3200	3239
OEND^b^		1998	2396	2802		3237	3606	3988
Safe opioid prescribing^c^		1666	1698	1707		2944	2976	2985
**Ohio**								
Sustain all 4 interventions	2084	3122	3945	4518	3724	5363	6513	7132
Increased MOUD initiation		2280	2409	2453		4356	4766	4904
Increased MOUD retention^a^		2269	2389	2437		4006	4154	4208
OEND^b^		2549	3032	3530		4140	4583	5052
Safe opioid prescribing^c^		2216	2280	2295		3856	3920	3936

Abbreviations: MOUD, medication for opioid use disorder; OEND, overdose education and naloxone distribution.

^a^ MOUD retention at the level observed in clinical trials (6-month retention of 46% for buprenorphine and 74% for methadone).

^b^ OEND that translates to a 10% mortality rate reduction.

^c^ An increase in safe opioid prescribing that translates to a 50% reduction in new prescription opioid misuse.

## Discussion

In this modeling analysis, we estimated reductions in OODs following the scaling up of different interventions and sustaining them for different durations in 4 states that have been highly impacted by the opioid overdose epidemic. We found that a substantial scale-up of MOUD initiation and retention, along with an increased supply of naloxone kits, would be needed in each state. Furthermore, sustaining interventions for a longer duration would be critical to achieving sustained reduction in OODs. Our study also found that once the interventions are stopped, the number of OODs is likely to start increasing again. For instance, positive gains associated with the implementation of EBPs for 2 years could be soon washed out if interventions are not sustained beyond the initial 2-year intervention period.

We acknowledge that implementing and sustaining interventions for a long period requires substantial resources. Sustaining all interventions was estimated to have the largest effect on the number of OODs; however, if only one intervention could be sustained for a longer duration, sustaining the increased supply of naloxone kits was estimated to prevent the largest number of OODs. In contrast, preventing prescription opioid misuse was estimated to have the smallest effect on OODs because most OODs are now caused by illicit opioids—not prescription opioids.^25^ Our analysis also highlights that any duration of sustainment of interventions is better than no sustainment. For instance, even 1-year sustainment of all interventions, beyond the initial period in Massachusetts, could reduce annual OODs by 21% to 37% vs 9% reduction without sustainment (Figure 4). Future studies could evaluate the cost-effectiveness of sustaining different types of interventions on the reduction in OODs.

Our study provides new insights to policy makers to address the ongoing opioid overdose crisis. An earlier modeling study by Linas and colleagues^45^ estimated the feasibility of reducing OODs in urban and rural communities in Massachusetts. Our analysis extends the scope of that study by including 4 states that have communities participating in the HEALing Communities Study and assessing the outcomes of sustaining interventions beyond the initial intervention period. For Massachusetts, our findings are in line with those of Linas et al,^45^ ie, reducing overdose deaths requires a substantial scale-up of MOUD initiation, MOUD retention, and naloxone use during the study period. Other modeling studies have evaluated the effect of interventions at the national level.^17,18,19,25,46^ While these studies provide important insights, the nature and scale of the opioid epidemic vary substantially from one state to another.^47^ We modeled the opioid overdose epidemic at the state level to capture the heterogeneous nature of the ongoing crisis. Although all 4 states included in our analysis are highly impacted, the prevalence of OUD and the scale of interventions at baseline (under current conditions) vary. As such, we observed variation in the effectiveness of outcomes. For instance, a 2-fold increase in MOUD initiation in Massachusetts had a higher reduction in OODs than other states, in particular Kentucky, because the proportion of individuals with OUD who are receiving MOUDs at baseline was higher in Massachusetts than in other states.

While our model is based on the HEALing Communities Study—one of the largest implementation studies—to evaluate the importance of sustained interventions to reduce OODs, the findings of our study are generalizable to other ongoing and future efforts to curb OOD trends. Sustaining interventions long term would require additional funding, infrastructure, and system-level changes. Without necessary resources, sustaining interventions would not be feasible, and we may not achieve substantial reductions in OODs in the United States.

### Limitations

Our study’s findings should be considered in the context of its assumptions and limitations. First, we modeled the impact of hypothetical increases in EBPs without explicitly modeling specific steps to achieve such increases in practice. For example, access to treatment can be limited by policy barriers, such as required prior authorization, limits for care, waiver requirements for buprenorphine prescribing, lack of opioid treatment programs where methadone can be administered, and/or challenges in accessing those programs, as most are located in urban communities.^48,49,50^ We did not account for those barriers to treatment and other factors that might make scaling up EBPs challenging. Second, because of lack of robust estimates of prevalence of opioid misuse and OUD, we used different data sources to indirectly estimate their prevalence. Third, while we accounted for interstate variability using publicly available state-level data, we could not account for the heterogeneity in the characteristics of the opioid overdose epidemic at the local level because of a lack of data at the community level.^51,52,53,54,55^ The impact of a combination of interventions for one community could differ from that of another. Fourth, we did not differentiate the pathways of people who are in recovery either because of non-MOUD treatment (eg, behavioral interventions) or no treatment. Fifth, we assumed that there was no interaction between interventions and that each intervention had an additive effect. Sixth, even though we incorporated the adverse impact of COVID-19 on the opioid epidemic, there are no data to inform the effect of COVID-19 on overdose mortality in the future. Therefore, based on recent trends,^37,38,39,40,41^ we assumed that the mortality rates would remain steady throughout the simulated time period.

## Conclusions

The findings of this study suggest that reducing opioid overdose deaths in Kentucky, Massachusetts, New York, and Ohio would require substantial scaling up of the delivery of medications for opioid use disorder and increasing the supply of naloxone. Sustained implementation of those interventions is needed to prevent opioid overdose deaths from rising again in those states.

## Notes

Supplement 1. **eAppendix 1.** Details of Dynamical Systems Model **eFigure 1.** Schematic of the Natural History for Nonmedical Opioid Use **eAppendix 2. **Model Parameter Estimation and Calibration **eFigure 2.** Linear Interpolations of Historical Buprenorphine and Methadone Treatment Counts **eTable 1.** Single-Year Calibration Targets by Year, 2015-2020 **eTable 2.** Multiyear Calibration Targets, 2015-2018 **eTable 3.** Model Calibration Targets and Corresponding Model Outputs **eFigure 3.** Distribution of Calibrated Model Parameter Values Over Their Search Ranges for Each State **eTable 4.** Estimates and Ranges of Calibrated Model Parameters **eFigure 4.** Simulation Band Created by the Mid-95th Percentile of the 1000 Calibrated Parameter Sets for Each State **eAppendix 3. **Parameters for Model Projection **eFigure 5.** Timeline Used in the Opioid Policy Model (OPyM) **eFigure 6. **Temporal Trends in Opioid Overdose Deaths Under Status Quo and Implementation of Interventions, With and Without Sustainment for Different Durations
eReferences.

Supplement 2.
Data Sharing Statement
