# Supplementary material for: Estimated Reductions in Opioid Overdose Deaths With Sustainment of Public Health Interventions in 4 US States
Source: JAMA Netw Open. 2023 Jun 9;6(6):e2314925. doi: 10.1001/jamanetworkopen.2023.14925 (PMC10257094; doi:10.1001/jamanetworkopen.2023.14925)
Supplement: Supplement 1. — eAppendix 1. Details of Dynamical Systems Model eFigure 1. Schematic of the Natural History for Nonmedical Opioid Use eAppendix 2. Model Parameter Estimation and Calibration eFigure 2. Linear Interpolations of Historical Buprenorphine and Methadone Treatment Counts eTable 1. Single-Year Calibration Targets by Year, 2015-2020 eTable 2. Multiyear Calibration Targets, 2015-2018 eTable 3. Model Calibration Targets and Corresponding Model Outputs eFigure 3. Distribution of Calibrated Model Parameter Values Over Their Search Ranges for Each State eTable 4. Estimates and Ranges of Calibrated Model Parameters eFigure 4. Simulation Band Created by the Mid-95th Percentile of the 1000 Calibrated Parameter Sets for Each State eAppendix 3. Parameters for Model Projection eFigure 5. Timeline Used in the Opioid Policy Model (OPyM) eFigure 6. Temporal Trends in Opioid Overdose Deaths Under Status Quo and Implementation of Interventions, With and Without Sustainment for Different Durations eReferences. [file jamanetwopen-e2314925-s001.pdf]

Supplemental Online Content

Chhatwal J, Mueller PP, Chen Q, et al. Estimated reductions in opioid overdose deaths with sustainment of public health interventions in 4 US states. *JAMA Netw Open.* 2023;6(6):e2314925. doi:10.1001/jamanetworkopen.2023.14925

eAppendix 1. Details of Dynamical Systems Model

eFigure 1. Schematic of the Natural History for Nonmedical Opioid Use

eAppendix 2. Model Parameter Estimation and Calibration

eFigure 2. Linear Interpolations of Historical Buprenorphine and Methadone Treatment Counts

eTable 1. Single-Year Calibration Targets by Year, 2015-2020

eTable 2. Multiyear Calibration Targets, 2015-2018

eTable 3. Model Calibration Targets and Corresponding Model Outputs

eFigure 3. Distribution of Calibrated Model Parameter Values Over Their Search Ranges for Each State

eTable 4. Estimates and Ranges of Calibrated Model Parameters

eFigure 4. Simulation Band Created by the Mid-95th Percentile of the 1000 Calibrated Parameter Sets for Each State

eAppendix 3. Parameters for Model Projection

eFigure 5. Timeline Used in the Opioid Policy Model (OPyM)

eFigure 6. Temporal Trends in Opioid Overdose Deaths Under Status Quo and Implementation of Interventions, With and Without Sustainment for Different Durations

eReferences.

This supplemental material has been provided by the authors to give readers additional information about their work.

© 2023 Chhatwal J et al. *JAMA Network Open.*

eAppendix 1. Details of Dynamical Systems Model

We developed a system dynamics model (also known as a *dynamical systems model* or *compartmental model*),[1] the ***O**pioid **P**olicy **M**odel (OPyM)*, to simulate the opioid crisis in Kentucky, Massachusetts, New York, and Ohio from 2015-2026 This model expands on previous works by Chen et al. 2019[2] by adding detailed treatment and relapse mechanics as well as expanding interventions to include harm reduction (naloxone), treatment initiation rates (buprenorphine and methadone), treatment retention rates, and the previously included opioid misuse prevention intervention. Our analysis focuses on the following key outcomes: opioid overdose deaths, opioid use disorder (OUD), and proportion of people on treatment.

We used a combination of data sources and model calibration to estimate the parameters in OPyM. State-specific opioid misuse incidence and prevalence as well as OUD prevalence came from the National Survey on Drug Use and Health (NSDUH).[3, 4] State-specific fatal opioid overdose data used multiple cause of death data from the Centers for Disease Control and Prevention (CDC).[5] Treatment for OUD is a complicated process and thus relied on several data sources. State-specific counts of people receiving medications for opioid use disorder (MOUD) used the Automated Reports and Consolidated Ordering System (ARCOS) for buprenorphine (BUP) data and the National Survey of Substance Abuse Treatment Services (N-SSATS) for methadone (MTD) data.[6, 7] Treatment retention and relapse rates used a combination of data from Timko et al. 2016, Morgan et al. 2018, and Ronquest et al. 2018.[8-10] Lastly, opioid overdose mortality rates for those on and off treatment depended on data from the systematic review and meta-analysis performed by Sordo et al. 2017.[11] The mortality rates on and off treatment were scaled by a calibrated parameter to allow for state-specific mortality rates. For model parameters not directly observable or that could not be informed by data, we relied on model calibration. Details on this calibration approach can be found in **eAppendix 2**.

In the remainder of this section, we describe the definition, structure, and evaluation of the system dynamics model in further detail, specifically:

1. Definition of compartments and OPyM's dynamical system of equations;
2. Expanding the traditional system dynamics model to emulate microsimulation model strengths.

S1.1 System Dynamics Model Structure

The Opioid Policy Model (OPyM) consists of 19 compartments that can be grouped into 4 main categories which represent the main stages of non-medical opioid use: prescription opioid misuse, illicit opioid use, opioid use disorder, and recovery. The following 19 compartments were adapted to the combination of data source definitions from the NSDUH and CDC data as well as medical experts to aid in accurately simulating opioid use disorder, treatment, and potential relapse (**eFigure 1**). See **eAppendix 2** for details on correcting NSDUH data to account for underestimates.

1. People with *prescription-only opioid misuse without an opioid use disorder*, denoted by compartment (**PN**). Estimates for the number of people in this compartment were based on NSDUH's individuals who claimed to use opioids non-medically in the past year, did not claim heroin use in the past year, and did not meet the DSM-IV criteria for opioid use disorder (OUD).
2. People with *illicit opioid use without an opioid use disorder*, denoted by compartment (**IN**). Estimates for the number of people in this compartment were based on NSDUH's individuals who claimed to use heroin in the past year but did not meet the DSM-IV criteria for OUD. It is worth noting that people without an OUD that used both prescription (Rx) and illicit opioids were assigned to this compartment as the potency of many illicit opioids would likely be the leading source of overdoses in the population.
3. People with an *opioid use disorder* can belong to one of two compartments (detailed below). Estimates for the total number of people in these 2 compartments were based on NSDUH's individuals who met the DSM-IV criteria for OUD.
    a. Active OUD, denoted by compartment (**OUD**). This represents individuals who did not recently relapse out of recovery, which could include those who never relapsed and/or arrived from compartments **PN** or **IN**.

    b. First month following relapse, denoted by compartment (**OUDrel**). This represents individuals who recently relapsed out of recovery back into having an OUD. Individuals that continue to have an OUD, beyond this first month, will be transferred into the **OUD** compartment.

4. People who are in *recovery* can follow one of many different paths through the following 15 compartments, split up into 3 subgroups: people on medication for opioid use disorder (MOUD) such as buprenorphine (BUP) and methadone (MTD), people who have discontinued MOUD but have not relapsed back to OUD, and people who are in recovery without the aid of medications (e.g., detox, residential programs, and psychosocial treatment).
    a. Individuals receiving MOUDs can be in 1 of 7 compartments, which represent durations of treatment. Details of how this was done without the need for a microsimulation or agent-based model can be found in **eAppendix1.2**.
        i. First month on MOUDs, denoted by compartment (**R1**). This represents individuals receiving either BUP or MTD for their first month of MOUD. Note that this includes those who may have been on treatment for several months previously, but who have relapsed and been off treatment for at least a month (our model's cycle length).
        ii. Second consecutive month on MOUDs, denoted by compartment (**R2**).
        iii. Third consecutive month on MOUDs, denoted by compartment (**R3**).
        iv. Fourth consecutive month on MOUDs, denoted by compartment (**R4**).
        v. Fifth consecutive month on MOUDs, denoted by compartment (**R5**).
        vi. Sixth consecutive month on MOUDs, denoted by compartment (**R6**).
        vii. Seven or more consecutive months on MOUDs, denoted by compartment (**R7p**).
    b. At any point of the above 7 compartments, individuals may either remain on treatment for another month, relapse to the **OUDrel** compartment, have a fatal overdose, die from other causes, or discontinue treatment without relapsing. Depending on how long an individual was on MOUDs prior to discontinuation without relapse (henceforth referred to as *discontinuation*), they may be less likely to relapse in the future. This is captured by the following 7 compartments.
        i. Discontinued after one month of treatment without relapse, denoted by compartment (**PR1**).
        ii. Discontinued after two consecutive months of treatment without relapse, denoted by compartment (**PR2**).
        iii. Discontinued after three consecutive months of treatment without relapse, denoted by compartment (**PR3**).
        iv. Discontinued after four consecutive months of treatment without relapse, denoted by compartment (**PR4**).
        v. Discontinued after five consecutive months of treatment without relapse, denoted by compartment (**PR5**).
        vi. Discontinued after six consecutive months of treatment without relapse, denoted by compartment (**PR6**).
        vii. Discontinued after seven or more consecutive months of treatment without relapse, denoted by compartment (**PR7p**).
    c. We assume that those who are off MOUDs must relapse before going back on MOUD.
    d. We lastly allow for people to enter recovery without the assistance of medication, denoted by compartment (**NMR**). This is not too frequent of an occurrence, normally requires some detox first, and is not a very successful form a treatment (even when combined with psychosocial treatments such as therapy, counseling, residential programs, or other methods).

eFigure 1. Schematic of the Natural History for Nonmedical Opioid Use

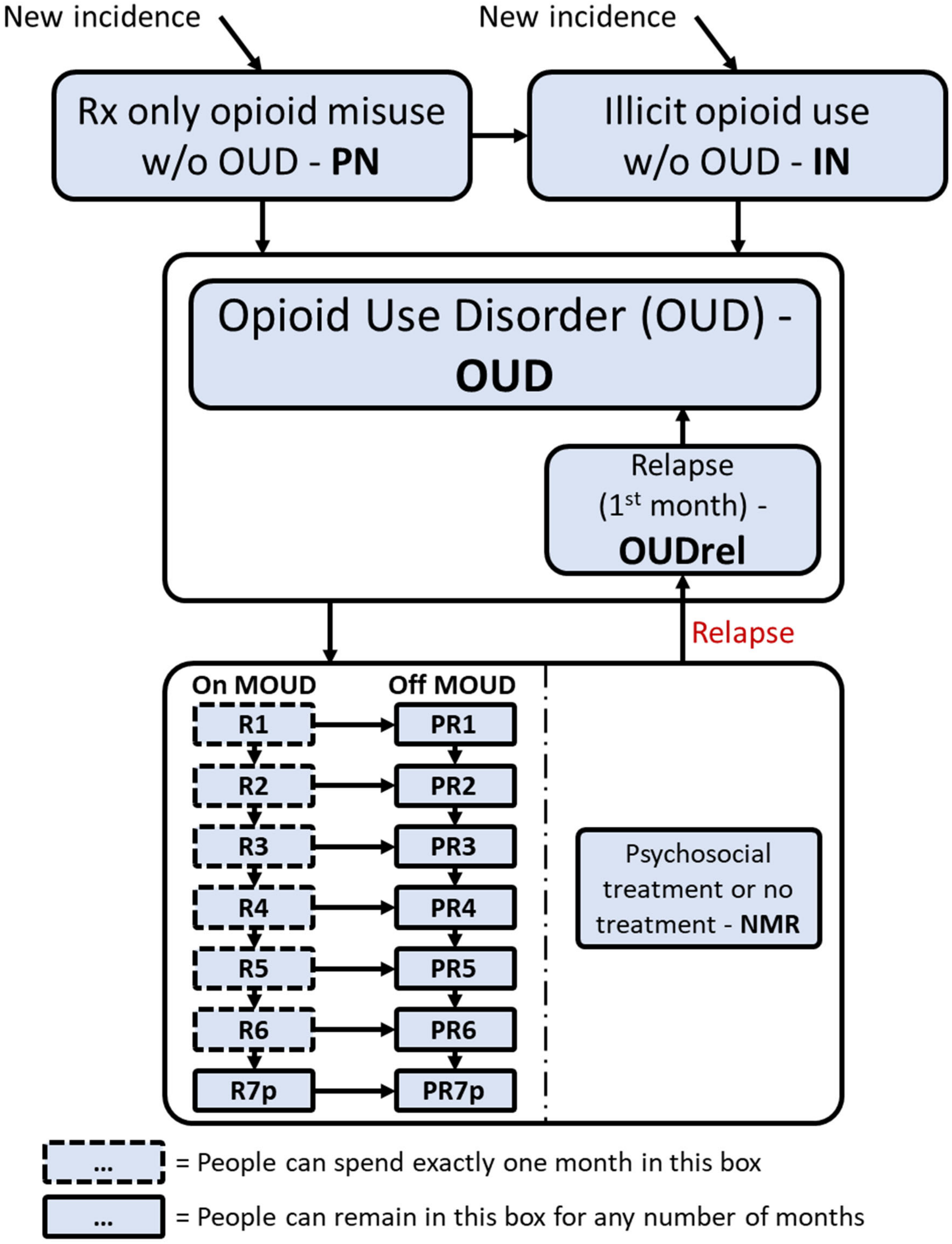

Mathematically, we used *PN(t), IN(t), OUD(t), OUDrel(t), R1(t), R2(t), …, R6(t), R7p(t), PR1(t), PR2(t), …, PR6(t), PR7p(t), and NMR(t)* to represent the number of people for each compartment at time *t*, and the model dynamics were represented by the following system of ordinary differential equations:

© 2023 Chhatwal J et al. *JAMA Network Open.*

$$\frac{dPN(t)}{dt} = \lambda_{PN} - \left(p_{PN\_OUD} + p_{PN\_IN} + \mu_{PN} + m_{PN}\right) \cdot PN(t),$$

$$\frac{dIN(t)}{dt} = \lambda_{IN}(t) + p_{PN\_IN} \cdot PN(t) - \left(p_{IN\_OUD} + \mu_{IN} + m_{IN}\right) \cdot IN(t),$$

$$\frac{dOUD(t)}{dt} = p_{PN\_OUD} \cdot PN(t) + p_{IN\_OUD} \cdot IN(t) + \frac{1}{T}\left(1 - T \cdot \left(p_{OUDrel\_R1} + p_{OUDrel\_NMR} + \mu_{OUDrel} + m_{PDrel} + m_{IDrel}(t)\right)\right) \cdot OUDrel(t),$$

$$\frac{dOUDrel(t)}{dt} = p_{R1\_OUDrel} \cdot R1(t) + p_{R2\_OUDrel} \cdot R2(t) + p_{R3\_OUDrel} \cdot R3(t) + p_{R4\_OUDrel} \cdot R4(t) + p_{R5\_OUDrel} \cdot R5(t) + p_{R6\_OUDrel} \cdot R6(t) + p_{R7p\_OUDrel} \cdot R7p(t) + p_{NMR\_OUDrel} \cdot NMR(t) - \frac{1}{T} \cdot OUDrel(t),$$

$$\frac{dR1(t)}{dt} = p_{OUD\_R1} \cdot OUD(t) + p_{OUDrel\_R1} \cdot OUDrel(t) - \frac{1}{T} \cdot R1(t),$$

$$\frac{dR2(t)}{dt} = p_{R1\_R2} \cdot R1(t) - \frac{1}{T} \cdot R2(t),$$

$$\frac{dR3(t)}{dt} = p_{R2\_R3} \cdot R2(t) - \frac{1}{T} \cdot R3(t),$$

$$\frac{dR4(t)}{dt} = p_{R3\_R4} \cdot R3(t) - \frac{1}{T} \cdot R4(t),$$

$$\frac{dR5(t)}{dt} = p_{R4\_R5} \cdot R4(t) - \frac{1}{T} \cdot R5(t),$$

$$\frac{dR6(t)}{dt} = p_{R5\_R6} \cdot R5(t) - \frac{1}{T} \cdot R6(t),$$

$$\frac{dR7p(t)}{dt} = p_{R6\_R7p} \cdot R6(t) - \frac{1}{T}\left(1 - \mathrm{T} \cdot \mathrm{p}_{\mathrm{R7p\_R7p}}\right) \cdot R7p(t),$$

$$\frac{\mathrm{dPR1(t)}}{\mathrm{dt}} = \frac{1}{\mathrm{T}}\left(1 - \mathrm{T} \cdot \left(\mathrm{p}_{\mathrm{R1\_R2}} + \mathrm{p}_{\mathrm{R1\_OUDrel}} + \mu_{\mathrm{R1}} + \mathrm{m}_{\mathrm{R1}}\right)\right) \cdot R1(\mathrm{t}) - \frac{1}{\mathrm{T}}\left(1 - \mathrm{T} \cdot \left(\mathrm{p}_{PR1\_\mathrm{OUDrel}} + \mu_{\mathrm{PR1}}\right)\right) \cdot PR1(\mathrm{t}),$$

$$\frac{\mathrm{dPR2(t)}}{\mathrm{dt}} = \frac{1}{\mathrm{T}}\left(1 - \mathrm{T} \cdot \left(\mathrm{p}_{\mathrm{R2\_R3}} + \mathrm{p}_{\mathrm{R2\_OUDrel}} + \mu_{\mathrm{R2}} + \mathrm{m}_{\mathrm{R2}}\right)\right) \cdot R2(\mathrm{t}) - \frac{1}{\mathrm{T}}\left(1 - \mathrm{T} \cdot \left(\mathrm{p}_{PR2\_\mathrm{OUDrel}} + \mu_{\mathrm{PR2}}\right)\right) \cdot PR2(\mathrm{t}),$$

$$\frac{\mathrm{dPR3(t)}}{\mathrm{dt}} = \frac{1}{\mathrm{T}}\left(1 - \mathrm{T} \cdot \left(\mathrm{p}_{\mathrm{R3\_R4}} + \mathrm{p}_{\mathrm{R3\_OUDrel}} + \mu_{\mathrm{R3}} + \mathrm{m}_{\mathrm{R3}}\right)\right) \cdot R3(\mathrm{t}) - \frac{1}{\mathrm{T}}\left(1 - \mathrm{T} \cdot \left(\mathrm{p}_{PR3\_\mathrm{OUDrel}} + \mu_{\mathrm{PR3}}\right)\right) \cdot PR3(\mathrm{t}),$$

$$\frac{\mathrm{dPR4(t)}}{\mathrm{dt}} = \frac{1}{\mathrm{T}}\left(1 - \mathrm{T} \cdot \left(\mathrm{p}_{\mathrm{R4\_R5}} + \mathrm{p}_{\mathrm{R4\_OUDrel}} + \mu_{\mathrm{R4}} + \mathrm{m}_{\mathrm{R4}}\right)\right) \cdot R4(\mathrm{t}) - \frac{1}{\mathrm{T}}\left(1 - \mathrm{T} \cdot \left(\mathrm{p}_{PR4\_\mathrm{OUDrel}} + \mu_{\mathrm{PR4}}\right)\right) \cdot PR4(\mathrm{t}),$$

$$\frac{\mathrm{dPR5(t)}}{\mathrm{dt}} = \frac{1}{\mathrm{T}}\left(1 - \mathrm{T} \cdot \left(\mathrm{p}_{\mathrm{R5\_R6}} + \mathrm{p}_{\mathrm{R5\_OUDrel}} + \mu_{\mathrm{R5}} + \mathrm{m}_{\mathrm{R5}}\right)\right) \cdot R5(\mathrm{t}) - \frac{1}{\mathrm{T}}\left(1 - \mathrm{T} \cdot \left(\mathrm{p}_{PR5\_\mathrm{OUDrel}} + \mu_{\mathrm{PR5}}\right)\right) \cdot PR5(\mathrm{t}),$$

$$\frac{\mathrm{dPR6(t)}}{\mathrm{dt}} = \frac{1}{\mathrm{T}}\left(1 - \mathrm{T} \cdot \left(\mathrm{p}_{\mathrm{R6\_R7}p} + \mathrm{p}_{\mathrm{R6\_OUDrel}} + \mu_{\mathrm{R6}} + \mathrm{m}_{\mathrm{R6}}\right)\right) \cdot R6(\mathrm{t}) - \frac{1}{\mathrm{T}}\left(1 - \mathrm{T} \cdot \left(\mathrm{p}_{PR6\_\mathrm{OUDrel}} + \mu_{\mathrm{PR6}}\right)\right) \cdot PR6(\mathrm{t}),$$

$$\frac{\mathrm{dPR7}p\mathrm{(t)}}{\mathrm{dt}} = \frac{1}{\mathrm{T}}\left(1 - \mathrm{T} \cdot \left(\mathrm{p}_{\mathrm{R7}p\mathrm{\_R7}p} + \mathrm{p}_{\mathrm{R7}p\mathrm{\_OUDrel}} + \mu_{\mathrm{R7p}} + \mathrm{m}_{\mathrm{R7p}}\right)\right) \cdot R7p(\mathrm{t}) - \left(p_{PR7p\_OUDrel} + \mu_{PR7p}\right) \cdot PR7p(\mathrm{t}),$$

$$\frac{dNMR(t)}{dt} = p_{OUD\_NMR} \cdot OUD(t) + p_{OUDrel\_NMR} \cdot OUDrel(t) - \left(p_{NMR\_OUDrel} + \mu_{NMR} + m_{NMR}\right) \cdot NMR(t) - \left(\frac{1}{2}\left(p_{OUD\_NMR} \cdot OUD(t) + p_{OUDrel\_NMR} \cdot OUDrel(t)\right) \cdot \left(1 - T \cdot p_{NMR\_OUDrel}\right)^{11}\right),$$

with initial conditions (PN(t_0), …) = (PN_0, …) and time scale $T = \frac{1}{12}$ used to scale annual rates and run our model in monthly time steps. Model variables are described as follows:

© 2023 Chhatwal J et al. *JAMA Network Open.*

- $PN, IN, OUD, OUDrel$: The prevalence of Rx-only opioid misuse without OUD (compartment PN), illicit opioid use without OUD (compartment IN), opioid use disorder – excluding people who relapsed in the past month (compartment OUD), and OUD with relapse in the past month (compartment OUDrel), respectively.
- $R1, R2, R3, R4, R5, R6, R7p$: The number of people who have been on MOUDs for less than 1 month (compartment R1), between 1 and 2 months (compartment R2), between 2 and 3 months (compartment R3), between 3 and 4 months (compartment R4), between 4 and 5 months (compartment R5), between 5 and 6 months (compartment R6), and for 6 or more months (compartment R7p), respectively.
- $PR1, PR2, PR3, PR4, PR5, PR6, PR7p$: The number of people who have discontinued MOUDs but remain in recovery (have not relapsed) and prior to discontinuing treatment, they were on MOUDs for less than 1 month (compartment PR1), between 1 and 2 months (compartment PR2), between 2 and 3 months (compartment PR3), between 3 and 4 months (compartment PR4), between 4 and 5 months (compartment PR5), between 5 and 6 months (compartment PR6), and for 6 or more months (compartment PR7p), respectively.
- NMR: The number of people who are in recovery without the assistance of MOUDs (compartment NMR).
- $PN_0, IN_0, OUD_0, OUDrel_0, R1_0, R2_0, \ldots, R6_0, R7p_0, PR1_0, PR2_0, \ldots, PR6_0, PR7p_0$: Initial value for each compartment at time t_0, i.e., year 2015.
- $\lambda_{PN}(s), \lambda_{IN}$: Annual incidence of Rx-only opioid misuse without OUD and of illicit opioid use without OUD (but not from Rx-only opioid misuse), respectively.
- $p_{PN\_IN}, p_{PN\_OUD}, p_{IN\_OUD}$: Annual transition rate from compartments *PN* to *IN*, *PN* to *OUD*, and *IN* to *OUD*, respectively.
- $p_{OUD\_R1}(s), p_{OUDrel\_R1}(s), p_{OUD\_NMR}, p_{OUDrel\_NMR}$: Annual transition rate from compartments *OUD* to *R1*, *OUDrel* to *R1*, *OUD* to *NMR*, and *OUDrel* to *NMR*, respectively. The first two can also be referred to as the MOUD initiation rate.
- $p_{R1\_R2}(s), p_{R2\_R3}(s), p_{R3\_R4}(s), p_{R4\_R5}(s), p_{R5\_R6}(s), p_{R6\_R7p}(s)$: Annual transition rate from compartments *R1* to *R2*, *R2* to *R3*, *R3* to *R4*, *R4* to *R5*, *R5* to *R6*, and *R6* to *R7p*, respectively. These can also be referred to as MOUD retention rates.
- $p_{R7p\_R7p}(s)$: Annual transition rate of remaining in compartment *R7p* for another cycle.
- $p_{R1\_OUDrel}(s), p_{R2\_OUDrel}(s), p_{R3\_OUDrel}(s), p_{R4\_OUDrel}(s), p_{R5\_OUDrel}(s), p_{R6\_OUDrel}(s), p_{R7p\_OUDrel}(s), p_{PR1\_OUDrel}, p_{PR2\_OUDrel}, p_{PR3\_OUDrel}, p_{PR4\_OUDrel}, p_{PR5\_OUDrel}, p_{PR6\_OUDrel}, p_{PR7p\_OUDrel}, p_{NMR\_OUDrel}$: Annual transition rate from compartments *R1* to *OUDrel*, *R2* to *OUDrel*, *R3* to *OUDrel*, *R4* to *OUDrel*, *R5* to *OUDrel*, *R6* to *OUDrel*, *R7p* to *OUDrel*, *PR1* to *OUDrel*, *PR2* to *OUDrel*, *PR3* to *OUDrel*, *PR4* to *OUDrel*, *PR5* to *OUDrel*, *PR6* to *OUDrel*, *PR7p*, and *NMR* to *OUDrel*, respectively. These can also be referred to as relapse rates.
- $m_{PN}(s), m_{IN}(s), m_{PD}(s), m_{ID}(s), m_{PDrel}(s), m_{IDrel}(s), m_{R1}(s), m_{R2}(s), m_{R3}(s), m_{R4}(s), m_{R5}(s), m_{R6}(s), m_{R7p}(s), m_{NMR}(s)$: Annual opioid overdose mortality rate from compartments *PN*, *IN*, *OUD* (attributed to Rx-only opioids), *OUD* (attributed to illicit opioids), *OUDrel* (attributed to Rx-only opioids), *OUDrel* (attributed to illicit opioids), *R1*, *R2*, *R3*, *R4*, *R5*, *R6*, *R7p*, and *NMR*, respectively.
- q_{R_odRx}: Proportion of overdose deaths from recovery that are attributed to Rx-only opioids.

© 2023 Chhatwal J et al. *JAMA Network Open.*

- μ_{PN}, μ_{IN}: Annual exit rate (consisting of other-cause mortality rate and opioid misuse cessation) from compartments *PN* and *IN*, respectively.
- $\mu_{OUD}, \mu_{OUDrel}, \mu_{R1}, \mu_{R2}, \dots, \mu_{R6}, \mu_{R7p}, \mu_{PR1}, \mu_{PR2}, \dots, \mu_{PR6}, \mu_{PR7p}, \mu_{NMR}$: Annual exit rate (consisting of other-cause mortality rate) from compartments *OUD*, *OUDrel*, *R1*, *R2*, *R3*, *R4*, *R5*, *R6*, *R7p*, *PR1*, *PR2*, *PR3*, *PR4*, *PR5*, *PR6*, *PR7p*, and *NMR*, respectively.

Abbreviations: Rx, prescription; OUD, opioid use disorder; MOUDs, medications for opioid use disorder.
Note: Parameters listed to be dependent on (s) are those that are adjusted either through interventions and/or COVID-19 impacts.

The system dynamics model was implemented in R programming language (version 4.2.0) using the "deSolve" package to solve the system of ordinary differential equations.

S1.2 Adapting System Dynamics Model to Track Months in Treatment

Instead of running our model in continuous time (as is most common for system dynamics or compartmental models), we ran our model with monthly time steps. Furthermore, we applied the Forward Euler method to OPyM's system of differential equations to enable us to create some compartments where people remain for precisely one month. An example of this application is creating sub-compartments for people who have been in treatment for varying number of months since the Forward Euler method can guarantee that the entire population that was in a given sub-compartment (e.g., **R2**) leaves that "health state" by the next time step by having exit rates sum to 100% (e.g., for **R2** the exit rates consist of being retained on treatment – moving to **R3**, discontinuing treatment – moving to **PR2**, having a fatal overdose, dying from another cause, or relapsing – moving to **OUDrel**).

© 2023 Chhatwal J et al. *JAMA Network Open.*

eAppendix 2. Model Parameter Estimation and Calibration

In this section, we describe the estimation and calibration of parameter values in our system dynamics model. To help overcome uncertainty in both model parameters and calibration targets, we performed 1,000 unique Simulated Annealing searches. From the 1,000 parameter sets we present the distributions of the input parameters (**eFigure 3** and **eTable 4**) and them plot both the mean and 95% confidence interval of the model outputs to display a "simulation band" that helps account for uncertainty in the model outcomes (e.g. **eFigure 4**).

S2.1 Parameter Estimation

We begin by elaborating on some calculations and adjustments made to data. The following subsections consist of correcting for underreporting in survey data, converting buprenorphine (BUP) distribution amounts to approximate number of people receiving BUP, recovering monthly retention and relapse rates from studies, and the impacts of COVID-19 on various model parameters.

Adjusting for underreporting of NSDUH data

Data from the National Survey on Drug Use and Health (NSDUH) is widely known to underestimate opioid misuse prevalence (for reasons such as self-reporting issues of surveys and underrepresented populations such as homeless or incarcerated people), but it is also one of the only sources to obtain this data. We resolve this issue by comparing NSDUH data to a capture-recapture analysis that more accurately estimates OUD prevalence.[12] Specifically, we derive a multiplier from comparing these two data sources as we cannot directly use the capture-recapture analysis due to the following reasons: it is only for the state of Massachusetts, it only covers the years 2011-2015, and it does not provide estimates for opioid misuse without OUD.[13]

From NSDUH we obtained the 2-year average OUD prevalence for Massachusetts covering 2015-2016: 54,995 people. To match this time window, we fit an exponential curve to the capture-recapture data from 2011 to 2015 and extrapolated it to get a 2016 estimate: 320,758 people. Then we averaged this value and the 2015 value (275,070) to get a 2-year average OUD prevalence of 297,914 people. The ratio of this value to the NSDUH estimate yields a multiplication scalar of 5.4171. We apply this multiplier to the OUD estimates from NSDUH across the 4 states and the years of data (see **eTable 2**).

We also expected the NSDUH data of opioid misuse without OUD to be underestimated. Without capture-recapture data of this population we had to make some assumptions. First, we recall that we divided people who misuse opioids without OUD into two compartments: people who only misuse prescription (Rx) opioids (**PN**) and people who use illicit opioids (with or without Rx opioids as well) (**IN**). We assumed that the prevalence of people who use illicit opioids without OUD is less of an underestimate than the OUD prevalence, but more so than that of the prevalence of people who misuse Rx opioids (only) without OUD. Specifically, we use 2/3^{rds} as much of a multiplier for people who use illicit opioids without OUD and 1/3rd as much for people who misuse Rx opioids (only) without OUD, or 3.9447 and 2.4724, respectively. It is worth noting that these multipliers cannot be obtained by directly multiplying 2/3^{rds} and 1/3rd to the OUD multiplier of 5.4171 because the lack of a multiplier is the same as a multiplier of 1, so instead we need to take that values that are 2/3^{rds} and 1/3rd along the interval between 1 and 5.4171.

Medications for opioid use disorder – treatment counts

We estimated the number of people receiving buprenorphine for the treatment of opioid use disorder (OUD) by using data obtained from the Automated Reports and Consolidated Ordering System (ARCOS). Specifically, the total grams of buprenorphine distributed per year were converted to the average number of doses of buprenorphine "distributed" per day using the following conversion factors:

- 365 or 366 days per year to determine the average grams of buprenorphine distributed per day

© 2023 Chhatwal J et al. *JAMA Network Open.*

- and a daily dosage of 0.016 grams per person to determine the average number of doses of buprenorphine distributed in a given day,

with the assumption of an average buprenorphine dosage of 16 mg per day. The number of doses "distributed" per day is then synonymous with the average number of people receiving a buprenorphine prescription, concurrently, for that year. We used ARCOS as a data source over various claims data due to both public availability and because states vary in the populations covered by claims data.

The amount of people receiving methadone for the treatment of OUD was obtained from the National Survey of Substance Abuse Treatment Services (N-SSATS). N-SSATS provides the number of people receiving methadone in a treatment facility on a single day, at the end of March of each year. We use this data as an annual average and calibrate by comparing it to the model output of March of each year.

Medications for opioid use disorder – retention and relapse rates

We begin this section by taking some time to clarify the semantics of retention and relapse. An individual who is currently receiving medications for opioid use disorder follows one of three possible paths (excluding overdose and other-cause mortality for simplicity): remain on treatment for another month or more (retention), discontinue treatment without relapse (transition to one of the **PR1** through **PR7p** health states), or discontinue treatment with relapse (relapse). Retention is the largest probability, further increasing the longer someone remains on treatment, ranging from 69.0% to 92.9% for buprenorphine. Relapse follows with the next largest probability, decreasing the longer someone remains on treatment, beginning at 27.0% the first month and decreasing down to 1.6% for those on buprenorphine for more than 6 months. Lastly, discontinuing treatment without relapse is on the order of a 5% monthly probability and is specifically derived as the leftover probability after accounting for mortality rates in addition to retention and relapse. We expand on the details and sources of these values in the following paragraphs.

Data on retention and relapse rates were more readily available for buprenorphine (BUP), so we begin with a focus on two studies about BUP. Data for retention rates is taken from Fig. 1 of Morgan et al. 2018, via the "Sublingual or oralmucosal buprenorphine/naloxone".[9] This data tracks the "time to medication discontinuation" and thus well approximates the probability of not being retained on treatment. We fit an exponential curve to this data, focus on the monthly values (due to the monthly cycle in our model), and take one minus these values to get the number of people retained on treatment over time. Lastly, we look at the ratio between consecutive months to determine the probability of remaining on treatment for at least one more month. The resulting probabilities are 68.0%, 82.0%, 85.1%, 86.9%, 88.1%, 89.0%, and 91.7%, for staying on treatment at least another month for those who have currently been on treatment for 1, 2, 3, 4, 5, 6, and 7 or more, consecutive months, respectively.

Relapse rates for BUP are estimated from data on indicators of relapse by Ronquest et al. 2018.[10] Data from this paper is presented in intervals of 20% of proportion of days covered (PDC). Data was taken from both the commercial and Medicaid samples of Table 3 in Ronquest et al. 2018. We assumed the midpoint of each PDC interval in terms of time to relapse (e.g., PDC<0.20 represented individuals who were on treatment for 1/10th of a year prior to relapse). As an example, we took the sum of "Any of the abovementioned relapse indicators" for those with PDC<0.20 from both the commercial and Medicaid samples – 1288 and 296, respectively – and divided by the total PDC<0.20 counts from the two samples – 4566 and 1519, respectively – to obtain a relapse probability of 26.0% for those who were on treatment for 1/10th of a year. After doing this for all 5 PDC groups, we fit an exponential curve to the data to calculate monthly relapse rates for the relevant MOUD compartments of our model. The resulting probabilities are 27.0%, 15.7%, 10.4%, 7.3%, 5.4%, 4.0%, and 1.6%, for the monthly probabilities of people who have been on treatment for 1, 2, 3, 4, 5, 6, and 7 or more, consecutive months, respectively.

Data on monthly retention and relapse rates for methadone (MTD) was not as readily available. Instead, we used data from a systematic review by Timko et al. that presented the average 6-month retention rates of BUP and MTD under randomized control trials, 46% and 74%, respectively [8]. The ratio of these retention rates under randomized control trials were used to scale the monthly retention rates of BUP found in Morgan et al., yielding 79.8%, 89.0%, 91.0%, 92.1%, 92.8%, 93.4%, and 95.0%, for staying on treatment at least another month for those who have

currently been on treatment for 1, 2, 3, 4, 5, 6, and 7 or more, consecutive months, respectively. We used the inverse relationship between BUP and MTD retention rates to scale down the relapse rates of BUP to get the relapse rates of MTD, yielding 17.0%, 9.5%, 6.2%, 4.4%, 3.2%, 2.4%, and 1.0%, for the monthly probabilities of people who have been on treatment for 1, 2, 3, 4, 5, 6, and 7 or more, consecutive months, respectively.

Due to data limitations, we did not create separate compartments in our model for people receiving buprenorphine versus those receiving methadone. However, we fit a trendline to both the ARCOS and N-SSATS data that was then used to create a state-specific, time-varying ratio of people on buprenorphine vs methadone (see **eFigure 2**). This ratio was then applied to get a weighted average of the overall retention and relapse rates.

© 2023 Chhatwal J et al. *JAMA Network Open.*

eFigure 2. Linear Interpolations of Historical Buprenorphine and Methadone Treatment Counts

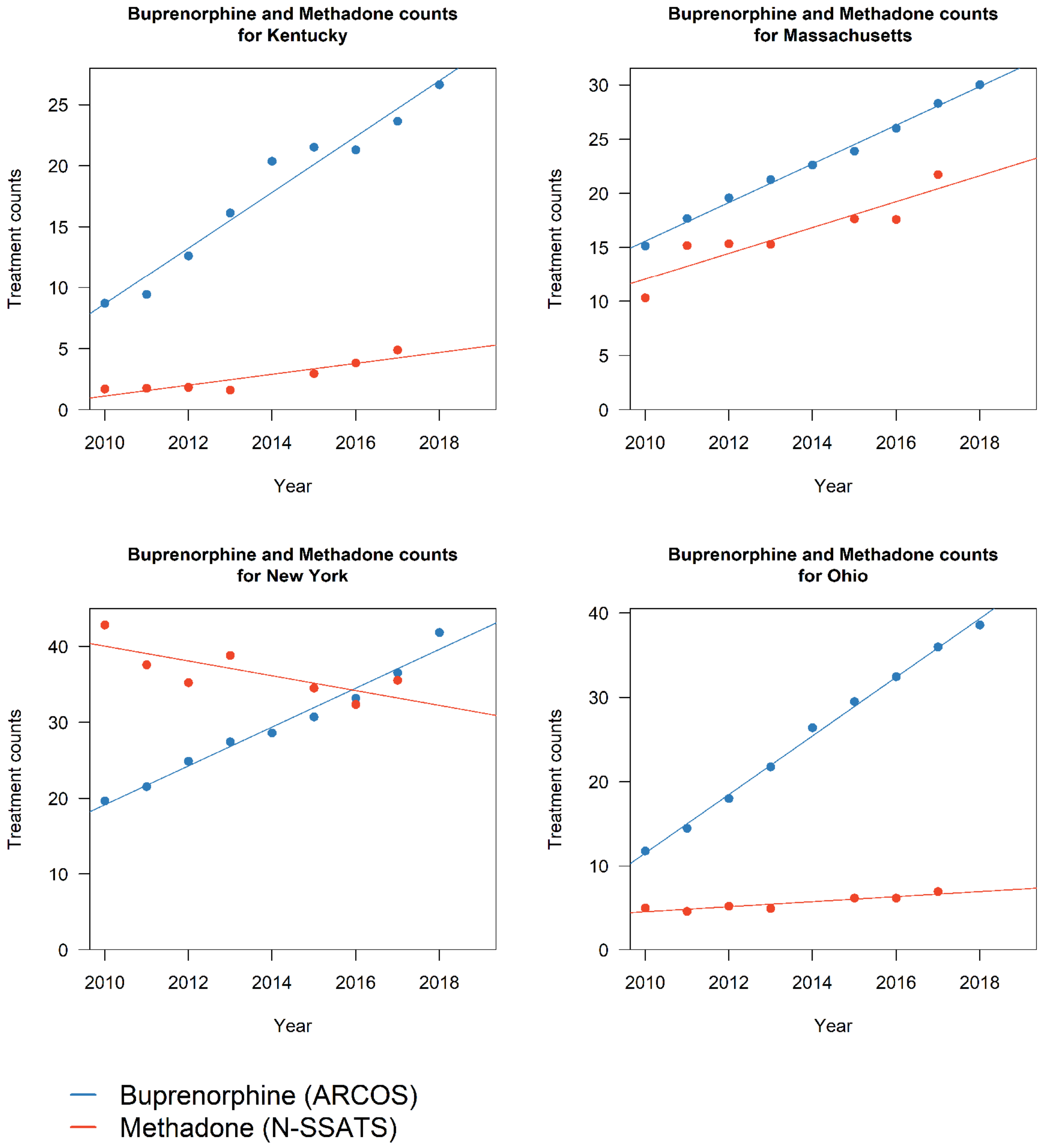

These are used to estimate the proportion of people on each medication during projections, which is necessary when averaging admission and retention rates.

Mortality rates while on treatment and immediately following relapse

One of the primary strengths of our model is having monthly compartments while on treatment to simulate the varying relapse rates based on treatment duration. We further built upon this by incorporating data from a systematic review and meta-analysis on the "mortality risk during and after opioid substitution treatment".[11] We primarily used data from the Kimber et al 2015 and Cousins et al 2016 studies due to their large sample size and that these studies

© 2023 Chhatwal J et al. *JAMA Network Open.*

presented results that covered our two most important results: how mortality rates change with duration on treatment and how mortality rates change following a relapse.

The relationship between overdose mortality rates and treatment duration come from Figure 6 of Sordo et al 2017. Averaging over treatments and study sizes yielded an annual overdose mortality rate of 0.0027 for people in the first 4 weeks of treatment and 0.0016 for people in treatment beyond 4 weeks. An exponential curve was fit to these data to obtain monthly overdose mortality rates for our 7 treatment compartments.

We also used Figure 6 of Sordo et al 2017 to determine the increased overdose risk following relapse by using the "out of treatment" columns. Averaging over the available data sets we found that people who relapsed in the past 4 weeks (OUDrel compartment) have a 75% higher overdose mortality rate than those who did not recently relapse (but who are also not currently on treatment – i.e., people in the OUD compartment).

Impact of COVID-19 on the opioid crisis

During the COVID-19 pandemic, we saw a significant increase in opioid overdose deaths which varied by state. The reasons for this increase in overdose deaths likely include: fentanyl penetrance continuing to increase regardless of COVID-19, more people using opioids alone, less bystanders around to witness overdoses, increased prevalence of opioid use disorder, comorbidities from increased alcohol use during the pandemic, and other mental health impacts of the opioid crisis.[14] Updated prevalence data was not available during any part of the pandemic, so we relied purely on overdose death data from CDC WONDER.[5]

In early 2020 we saw an increase in overdose deaths which was sustained all the way through 2021 and beyond. Specifically, our model assumes an increase in overdose mortality rates in all compartments beginning in January 2020, gradually increasing to its full impact by March 2020, and then remained high for the duration of our projections. The overdose mortality rates in each compartment were scaled by a multiplier with the values of 1.6, 1.05, 1.5, and 1.25, for Kentucky, Massachusetts, New York, and Ohio, respectively. The United States as a whole saw a multiplier of 1.5, which will be relevant to the next paragraph on how the pandemic impacted medications for opioid use disorder.

The pandemic also stressed the United States healthcare system in many ways. Literature suggests that treatment initiation rates were reduced by approximately 28% in the first few months of 2020 and returned to about 90% of pre-pandemic rates by June 2020.[15, 16] Due to lack of state-specific data, we assumed these rates applied to the entire nation and use the ratio of state-specific overdose death rate multipliers to the national-level multiplier in the previous paragraph to obtain COVID-related changes in MOUD initiation by state. As a result, treatment initiation rates reach a low point of 66.4%, 97.2%, 72%, and 86% when compared to pre-pandemic rates, and bounced back, mostly, to 88%, 99%, 90%, and 95% of pre-pandemic rates for Kentucky, Massachusetts, New York, and Ohio, respectively.

Data does not suggest that the COVID-19 pandemic significantly affected retention rates for those already receiving medications for opioid use disorder.[15]

S2.2 Model Calibration

Not all model parameters can be estimated from data. This is due to a combination of either a lack of data or transition rates that are not able to be measured directly from existing data. In the following sections we elaborate on the various calibration details, including calibration parameters, calibration targets, goodness-of-fit measure, search algorithm, and the calibration results.

Calibration parameters

A total of 20 model parameters were determined by model calibration, which are defined below:

© 2023 Chhatwal J et al. *JAMA Network Open.*

- $PN_0, IN_0, (OUD_0 + OUDrel_0), R_0$: initial sizes of compartments PN, IN, (OUD + OUDrel), and all recovery compartments combined, respectively, where the latter 2 values are distributed amongst their respective compartments by assuming a steady state was reached prior to simulation (4 parameters),
- $\lambda_{PN}, \lambda_{IN}$: incidence rate into compartments PN and IN, respectively, with both a baseline value and an annual percent change (APC) for the latter (3 parameters),
- $p_{PN\_IN}, p_{PN\_OUD}, p_{IN\_OUD}, p_{OUD\_R1}$: transition rate from compartment PN to IN, PN to OUD, IN to OUD, and OUD to R1, respectively (4 parameters),
- μ_{PN}, μ_{IN}: exit rate (consisting of cessation of opioid misuse and other-cause mortalities) from compartments PN and IN, respectively (2 parameters),
- $m_{PN}, m_{IN}, m_{PD}, m_{ID}$: opioid overdose mortality rate for compartments PN, IN, OUD - attributed to Rx opioids only, and OUD - attributed to illicit opioids, respectively, with both at baseline and APC value for m_{ID} (5 parameters),
- q_{R_odRx}: proportion of opioid overdose deaths from people on treatment that are attributed to Rx opioids only (1 parameter),
- s_{odMOUD}: scalar used to create state-specific opioid overdose mortality rates while on treatment (1 parameter).

The final calibration parameter is used to bridge the disconnect between state-specific opioid overdose rates from people with an active opioid use disorder (OUD and not on medications) and non-state-specific opioid overdose rates while on treatment. In short, it is applied as a multiplier to the values derived from Sordo et al 2017 (see **Section S2.1**).

Calibration targets

We compared OPyM's outcomes with data from the following sources: NIDA/NSDUH for Rx-only opioid misuse prevalence without opioid use disorder (OUD), illicit opioid use prevalence without OUD, OUD prevalence, and new incidences of Rx-only opioid misuse without OUD;[3, 4] a combination of ARCOS and N-SSATS for the average number of individuals receiving medications for OUD;[6, 7] and CDC WONDER data for the number of total opioid overdose deaths as well as illicit opioid overdose deaths (with or without Rx opioids as a multiple cause of death) (**eTable 1** and **eTable 2**). [5]

© 2023 Chhatwal J et al. *JAMA Network Open.*

eTable 1. Single-Year Calibration Targets by Year, 2015-2020

	Year	Average annual individuals receiving medications for opioid use disorder (compartments R1 through R7p)	Number of overdose deaths from all opioids	Number of overdose deaths from illicit opioids
**State: KY**				
	2015	24,476	883	596
	2016	25,133	988	714
	2017	28,540	1,160	934
	2018	NA	989	818
	2019	NA	1,036	875
	2020	NA	1,686	1,531
**State: MA**				
	2015	41,532	1,549	1,418
	2016	43,604	1,990	1,836
	2017	50,046	1,913	1,789
	2018	NA	1,990	1,879
	2019	NA	1,969	1,896
	2020	NA	2,064	1,965
**State: NY**				
	2015	65,271	2,162	1,588
	2016	65,567	3,005	2,431
	2017	72,074	3,220	2,737
	2018	NA	2,987	2,521
	2019	NA	2,934	2,570
	2020	NA	4,230	3,872
**State: OH**				
	2015	35,654	2,695	2,269
	2016	38,579	3,610	3,159
	2017	42,885	4,286	3,879
	2018	NA	3,234	2,979
	2019	NA	3,444	3,253
	2020	NA	4,380	4,191

© 2023 Chhatwal J et al. *JAMA Network Open.*

eTable 2. Multiyear Calibration Targets, 2015-2018

	Year	Incidence of Rx-only opioid misuse without OUD*	Prevalence of Rx-only opioid misuse without OUD* (compartment PN)	Prevalence of illicit opioid use without OUD* (compartment IN)	Prevalence of opioid use disorder*
**State: KY**					
	2015-2016	75,771	315,830	17,170	336,242
	2016-2017	41,872	280,323	3,207	271,983
	2017-2018	31,898	277,372	14,543	300,384
	-	-	-	-	-
	2016-2018	42,474	277,459	11,833	303,036
**State: MA**					
	2015-2016	109,916	413,283	16,320	297,914
	2016-2017	129,924	303,078	50,890	382,560
	2017-2018	105,928	304,488	91,877	367,568
	-	-	-	-	-
	2016-2018	123,048	322,761	67,306	332,956
**State: NY**					
	2015-2016	257,974	1,270,169	82,755	568,369
	2016-2017	195,869	1,066,832	42,749	607,884
	2017-2018	136,132	918,825	10,522	522,441
	-	-	-	-	-
	2016-2018	173,080	981,687	33,421	553,174
**State: OH**					
	2015-2016	195,860	918,799	8,401	448,217
	2016-2017	140,915	947,231	39,060	547,251
	2017-2018	143,985	745,007	64,103	743,081
	-	-	-	-	-
	2016-2018	144,957	813,780	42,735	633,791

*The above calibration targets represent the scaled-up values (using the NSDUH multipliers)

Goodness-of-fit metric

For each set of model parameters, we assessed how well the model matched to the calibration targets by comparing the model outputs O_i with corresponding calibration targets E_i (**eTable 3**). To account for the wide variety of calibration target units and/or scales, we normalized the sum of squared errors between model outputs and calibration targets by the average value of the given target, $\bar{E}_i$. The overall goodness-of-fit measure, the *total error*, was defined as the summation of model errors over all calibration targets (with equal weights):

$$\text{total error} = \sum_i \sum_{t=t_1}^{t_n} \left(\frac{E_i(t) - O_i(t)}{\bar{E}_i(t)} \times 100 \right)^2.$$

© 2023 Chhatwal J et al. *JAMA Network Open.*

eTable 3. Model Calibration Targets and Corresponding Model Outputs

Index (i)	Calibration target (E_i)	Model output (O_i)
1	Incidence of prescription (Rx) opioid misuse without opioid use disorder (OUD)	$\lambda_{PN}(t)$
**2**	Prevalence of prescription (Rx) opioid misuse without opioid use disorder (OUD)	$PN(t)$
3	Prevalence of illicit opioid use without OUD*	$IN(t)$
4	Prevalence of OUD – note that data from NSDUH is based on survey questions about the past 12 months, so we consider also include people who have been on treatment for less than 12 months	$OUD(t) + OUDrel(t) + R1(t) + \cdots + R6(t) + \left(1-(1-p_{R7p\_OUDrel})^5\right) \cdot R7p(t) + \left(1-(1-p_{PR1\_OUDrel})^{10}\right) \cdot PR1(t) + \left(1-(1-p_{PR2\_OUDrel})^9\right) \cdot PR2(t) + \left(1-(1-p_{PR3\_OUDrel})^8\right) \cdot PR3(t) + \left(1-(1-p_{PR4\_OUDrel})^7\right) \cdot PR4(t) + \left(1-(1-p_{PR5\_OUDrel})^6\right) \cdot PR5(t) + \left(1-(1-p_{PR6\_OUDrel})^5\right) \cdot PR6(t) + \left(1-(1-p_{PR7p\_OUDrel})^4\right) \cdot PR7p(t) + \left(1-(1-p_{NMR\_OUDrel})^{11}\right) \cdot NMR(t)$
5	Average annual individuals receiving medications for OUD	$R1(t) + R2(t) + R3(t) + R4(t) + R5(t) + R6(t) + R7p(t)$
6	Overdose death from all opioids (from all compartments)	$m_{PN} \cdot PN(t) + m_{IN} \cdot IN(t) + \left(m_{PD} + m_{ID}(t)\right) \cdot OUD(t) + \left(m_{PDrel} + m_{IDrel}(t)\right) \cdot OUDrel(t) + m_{R1} \cdot R1(t) + \cdots + m_{R7p} \cdot R7p(t) + m_{PR1} \cdot PR1(t) + \cdots + m_{PR7p} \cdot PR7p(t) + m_{NMR} \cdot NMR(t)$
7	Overdose death from illicit opioids (from all relevant compartments)	$m_{IN} \cdot IN(t) + m_{ID}(t) \cdot OUD(t) + m_{IDrel}(t) \cdot OUDrel(t) + \left(1 - q_{R\_odRx}\right)\left(m_{R1} \cdot R1(t) + \cdots + m_{R7p} \cdot R7p(t) + m_{PR1} \cdot PR1(t) + \cdots + m_{PR7p} \cdot PR7p(t) + m_{NMR} \cdot NMR(t)\right)$

*Due to data suppression, this calibration target was often an underestimate and thus for years when E_3 was smaller than O_3, an error of zero was used.

Calibration algorithm

To calibrate our model, we used the Generalized Simulated Annealing search algorithm from the package "GenSA" with stopping conditions of either 500 maximum iterations or 1 hour on a computing cluster – ERISTwo at Mass General Brigham. For each state we performed a total of 1,000 independent searches with a starting search location determined by 1,000 points on a Latin Hypercube in 20-dimensional space (across our 20 calibrated parameters and their ranges).

Calibration results

To account for uncertainty in our model parameters, we used all 1,000 parameter sets for each state to run simulations – with and without interventions, this results in a "simulation band" around outcomes (see **eFigure 4**).

© 2023 Chhatwal J et al. *JAMA Network Open.*

In the main manuscript, these simulation bands are often suppressed for visual clarity, and instead the average of the outcomes is shown as one line (as opposed to the outcome of the average parameter set).

eFigure 3. Distribution of Calibrated Model Parameter Values Over Their Search Ranges for Each State

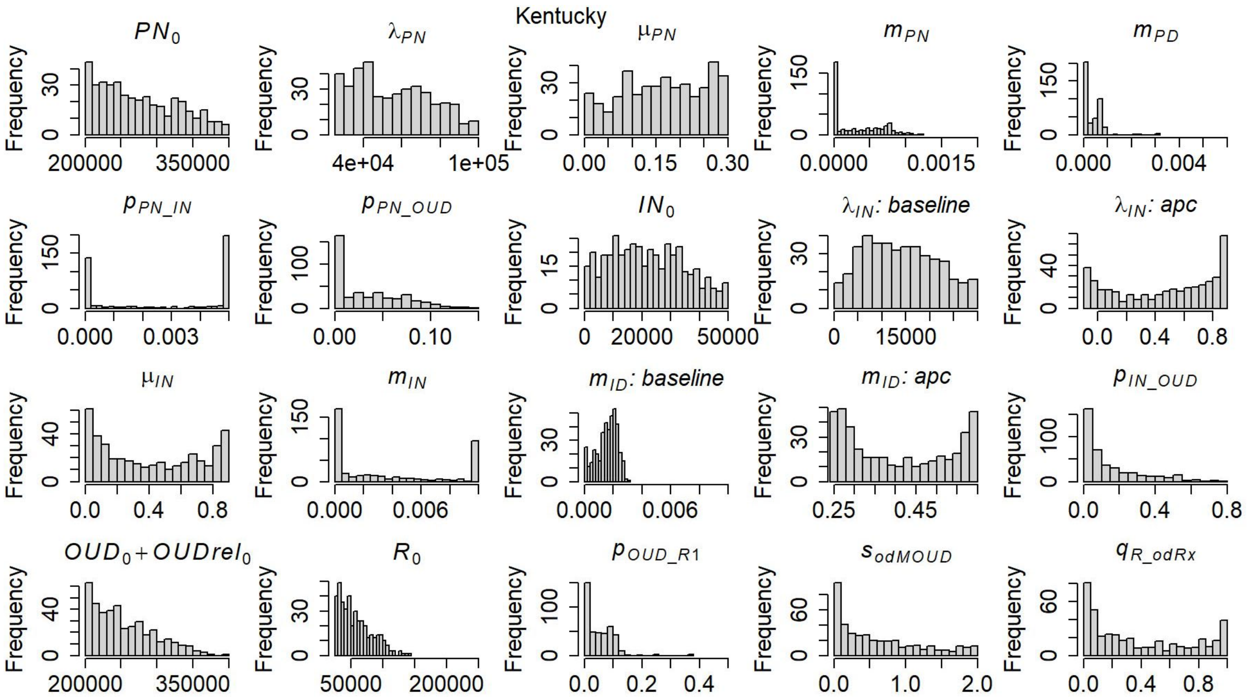

eFigure 3a. Distribution of calibrated model parameter values over their search ranges – Kentucky.

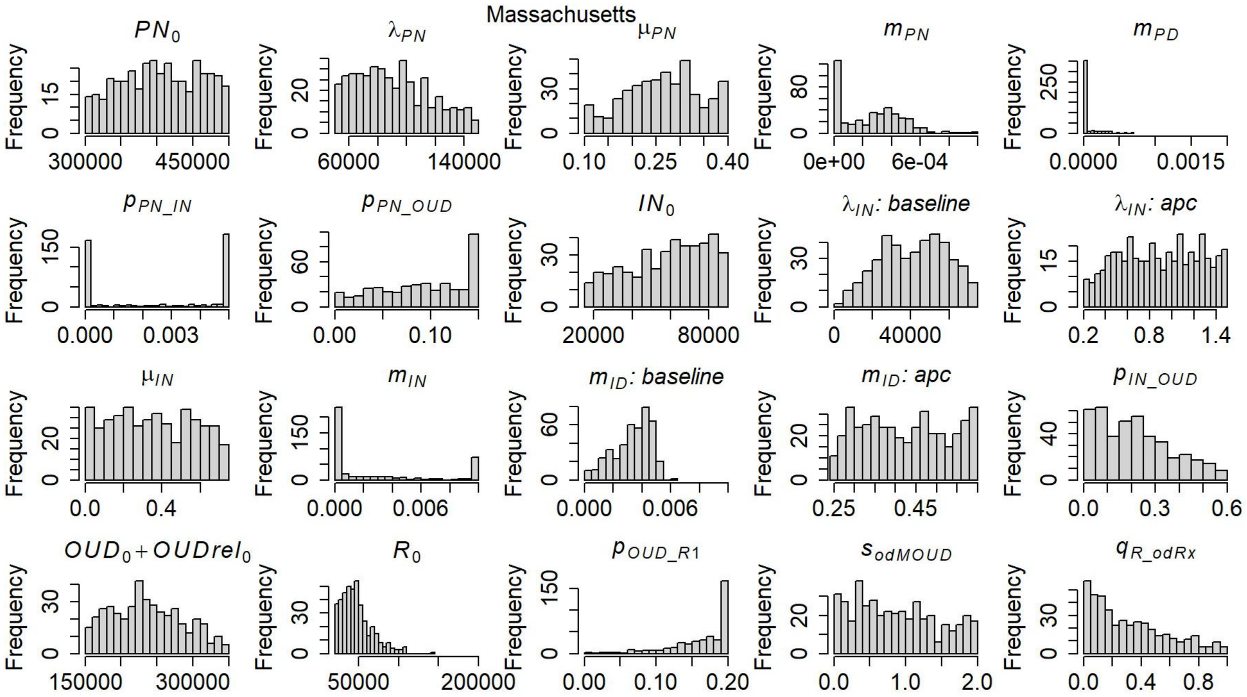

eFigure 3b. Distribution of calibrated model parameter values over their search ranges – Massachusetts.

© 2023 Chhatwal J et al. *JAMA Network Open.*

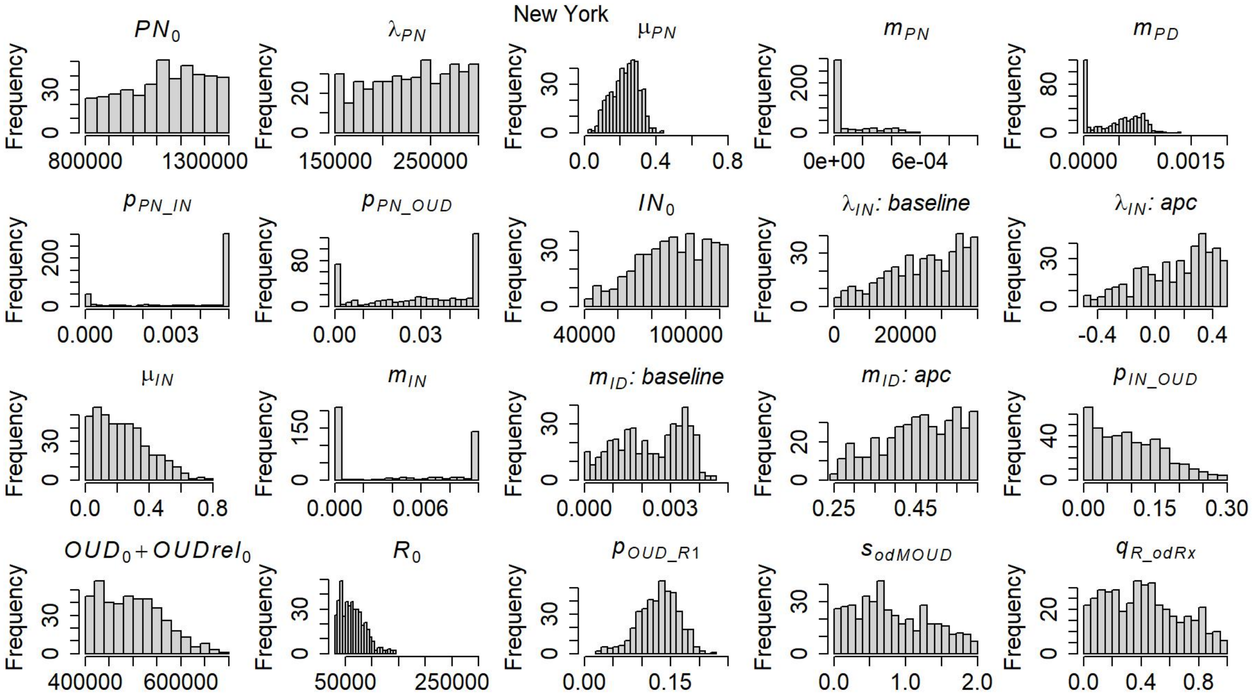

eFigure 3c. Distribution of calibrated model parameter values over their search ranges – New York.

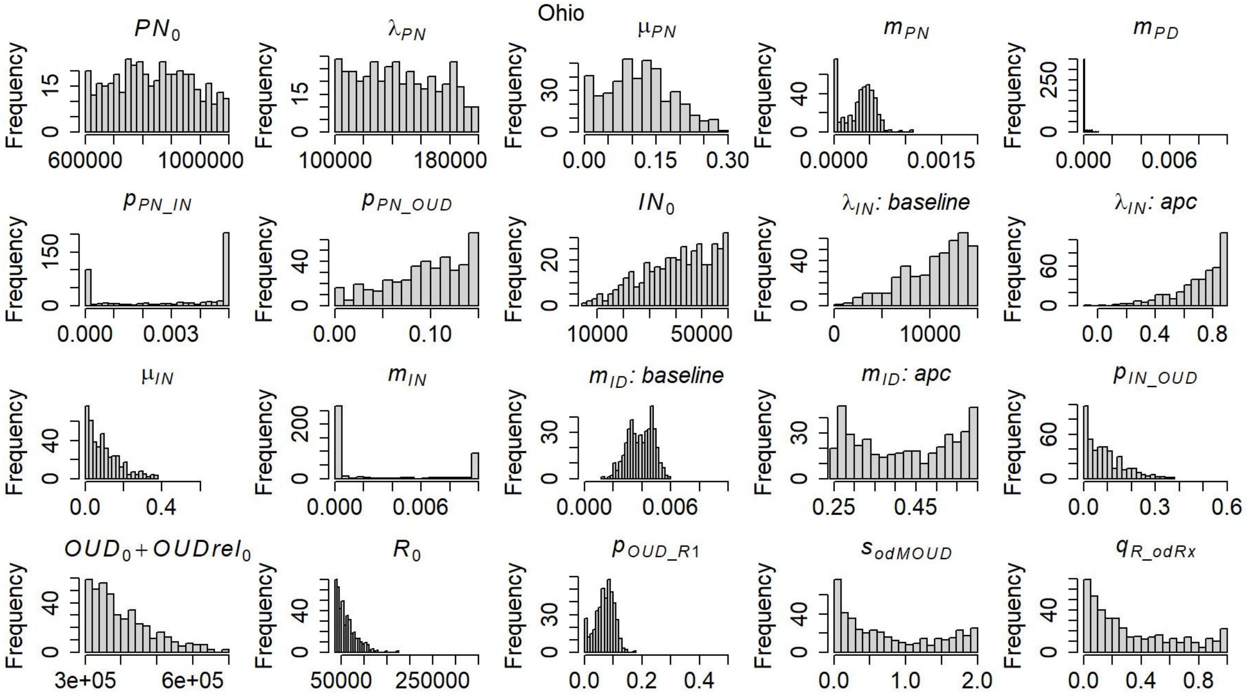

eFigure 3d. Distribution of calibrated model parameter values over their search ranges – Ohio.

© 2023 Chhatwal J et al. *JAMA Network Open.*

eTable 4. Estimates and Ranges of Calibrated Model Parameters

KY Parameters	Value (SD)	Interquartile Range
Prevalence of Rx-only opioid misuse without OUD in 2015, PN_0	275188 (53891)	229217 - 320460
Prevalence of illicit opioid use without OUD in 2015, IN_0	22030 (12907)	11522 - 31930
Prevalence of OUD (excluding people who relapsed in the past month) and OUD with relapse in the past month in 2015, $OUD_0 + OUDrel_0$	253946 (41966)	218153 - 280950
Number of people in recovery in 2015, R_0	57733 (25048)	36894 - 72859
Annual incidence of Rx-only opioid misuse without OUD, λ_{PN}	55101 (19643)	38725 - 70254
Annual incidence of illicit opioid use without OUD (but not from Rx-only opioid misuse) at baseline, λ_{IN}: baseline	14021 (7504)	7709 - 19657
Annual percentage change in incidence of illicit opioid use without OUD (but not from Rx-only opioid misuse), λ_{IN}: apc	0.467 (0.346)	0.114 - 0.788
Annual transition rate from compartments *PN* to *IN*, p_{PN_IN}	0.00287 (0.00233)	0 - 0.005
Annual transition rate from compartments *PN* to *OUD*, p_{PN_OUD}	0.0332 (0.0351)	0 - 0.0579
Annual transition rate from compartments *IN* to *OUD*, p_{IN_OUD}	0.147 (0.169)	0.0205 - 0.214
Annual transition rate from compartments *OUD* to *R1*, p_{OUD_R1}	0.0489 (0.0494)	0 - 0.0854
Annual exit rate from compartment *PN*, μ_{PN}	0.163 (0.086)	0.0939 - 0.24
Annual exit rate from compartment *IN*, μ_{IN}	0.408 (0.313)	0.104 - 0.704
Annual opioid overdose mortality rate from compartment *PN*, m_{PN}	0.000311 (0.000337)	0 - 0.000614
Annual opioid overdose mortality rate from compartment *IN*, m_{IN}	0.0036 (0.00408)	0 - 0.00813
Annual opioid overdose mortality rate from compartment *OUD* (attributed to Rx-only opioids), m_{PD}	0.000334 (0.000399)	0 - 0.000638
Annual opioid overdose mortality rate from compartment *OUD* (attributed to illicit opioids) at baseline, m_{ID}: baseline	0.00158 (0.00072)	0.00114 - 0.00214
Annual percentage change in opioid overdose mortality rate from compartment *OUD* (attributed to illicit opioids), m_{ID}: apc	0.407 (0.126)	0.283 - 0.535
Proportion of overdose deaths from recovery that are attributed to Rx-only opioids, q_{R_odRx}	0.383 (0.348)	0.0713 - 0.698
Scalar for opioid overdose mortality rate while on treatment, s_{odMOUD}	0.611 (0.582)	0.114 - 0.966

MA Parameters	Value (SD)	Interquartile Range
Prevalence of Rx-only opioid misuse without OUD in 2015, PN_0	404885 (54300)	362389 - 455054
Prevalence of illicit opioid use without OUD in 2015, IN_0	58201 (20552)	42733 - 75397

Prevalence of OUD (excluding people who relapsed in the past month) and OUD with relapse in the past month in 2015, $OUD_0 + OUDrel_0$	237043 (48875)	197384 - 273305
Number of people in recovery in 2015, R_0	47141 (19344)	33400 - 56817
Annual incidence of Rx-only opioid misuse without OUD, λ_{PN}	90906 (25705)	69789 - 110917
Annual incidence of illicit opioid use without OUD (but not from Rx-only opioid misuse) at baseline, λ_{IN}: baseline	41851 (17126)	28133 - 55759
Annual percentage change in incidence of illicit opioid use without OUD (but not from Rx-only opioid misuse), λ_{IN}: apc	0.887 (0.359)	0.589 - 1.19
Annual transition rate from compartments *PN* to *IN*, p_{PN_IN}	0.00263 (0.00235)	0 - 0.005
Annual transition rate from compartments *PN* to *OUD*, p_{PN_OUD}	0.0921 (0.0461)	0.0541 - 0.137
Annual transition rate from compartments *IN* to *OUD*, p_{IN_OUD}	0.213 (0.15)	0.081 - 0.311
Annual transition rate from compartments *OUD* to *R1*, p_{OUD_R1}	0.164 (0.0432)	0.143 - 0.2
Annual exit rate from compartment *PN*, μ_{PN}	0.265 (0.0778)	0.211 - 0.319
Annual exit rate from compartment *IN*, μ_{IN}	0.356 (0.213)	0.175 - 0.544
Annual opioid overdose mortality rate from compartment *PN*, m_{PN}	0.000253 (0.000215)	0 - 0.000408
Annual opioid overdose mortality rate from compartment *IN*, m_{IN}	0.00268 (0.00384)	0 - 0.0042
Annual opioid overdose mortality rate from compartment *OUD* (attributed to Rx-only opioids), m_{PD}	3.67e-05 (9.7e-05)	0 - 0
Annual opioid overdose mortality rate from compartment *OUD* (attributed to illicit opioids) at baseline, m_{ID}: baseline	0.00337 (0.00128)	0.00267 - 0.00439
Annual percentage change in opioid overdose mortality rate from compartment *OUD* (attributed to illicit opioids), m_{ID}: apc	0.425 (0.104)	0.335 - 0.511
Proportion of overdose deaths from recovery that are attributed to Rx-only opioids, q_{R_odRx}	0.321 (0.26)	0.102 - 0.486
Scalar for opioid overdose mortality rate while on treatment, s_{odMOUD}	0.873 (0.572)	0.378 - 1.3

NY Parameters	Value (SD)	Interquartile Range
Prevalence of Rx-only opioid misuse without OUD in 2015, PN_0	1133522 (165236)	1001123 - 1276101
Prevalence of illicit opioid use without OUD in 2015, IN_0	91739 (20603)	77491 - 108572
Prevalence of OUD (excluding people who relapsed in the past month) and OUD with relapse in the past month in 2015, $OUD_0 + OUDrel_0$	498489 (64295)	444270 - 542384
Number of people in recovery in 2015, R_0	64064 (23053)	44546 - 78333

© 2023 Chhatwal J et al. *JAMA Network Open.*

Annual incidence of Rx-only opioid misuse without OUD, λ_{PN}	230203 (43283)	195549 - 269211
Annual incidence of illicit opioid use without OUD (but not from Rx-only opioid misuse) at baseline, λ_{IN}: baseline	25136 (10370)	17406 - 34330
Annual percentage change in incidence of illicit opioid use without OUD (but not from Rx-only opioid misuse), λ_{IN}: apc	0.137 (0.252)	-0.3997
Annual transition rate from compartments *PN* to *IN*, p_{PN_IN}	0.00399 (0.00183)	0.00402 - 0.005
Annual transition rate from compartments *PN* to *OUD*, p_{PN_OUD}	0.0302 (0.0188)	0.0143 - 0.05
Annual transition rate from compartments *IN* to *OUD*, p_{IN_OUD}	0.098 (0.0714)	0.0361 - 0.148
Annual transition rate from compartments *OUD* to *R1*, p_{OUD_R1}	0.129 (0.0344)	0.106 - 0.153
Annual exit rate from compartment *PN*, μ_{PN}	0.228 (0.0739)	0.175 - 0.284
Annual exit rate from compartment *IN*, μ_{IN}	0.237 (0.164)	0.101 - 0.338
Annual opioid overdose mortality rate from compartment *PN*, m_{PN}	8.74e-05 (0.000149)	0 - 0.000126
Annual opioid overdose mortality rate from compartment *IN*, m_{IN}	0.00435 (0.00461)	0 - 0.01
Annual opioid overdose mortality rate from compartment *OUD* (attributed to Rx-only opioids), m_{PD}	0.000437 (0.00034)	5e-06 - 0.000741
Annual opioid overdose mortality rate from compartment *OUD* (attributed to illicit opioids) at baseline, m_{ID}: baseline	0.0023 (0.00116)	0.00138 - 0.00335
Annual percentage change in opioid overdose mortality rate from compartment *OUD* (attributed to illicit opioids), m_{ID}: apc	0.457 (0.0924)	0.391 - 0.537
Proportion of overdose deaths from recovery that are attributed to Rx-only opioids, q_{R_odRx}	0.422 (0.258)	0.202 - 0.612
Scalar for opioid overdose mortality rate while on treatment, s_{odMOUD}	0.826 (0.523)	0.425 - 1.25

OH Parameters	Value (SD)	Interquartile Range
Prevalence of Rx-only opioid misuse without OUD in 2015, PN_0	838576 (134563)	735942 - 946720
Prevalence of illicit opioid use without OUD in 2015, IN_0	40578 (13193)	31028 - 51219
Prevalence of OUD (excluding people who relapsed in the past month) and OUD with relapse in the past month in 2015, $OUD_0 + OUDrel_0$	404160 (85425)	337661 - 450949
Number of people in recovery in 2015, R_0	59721 (21377)	43378 - 70792
Annual incidence of Rx-only opioid misuse without OUD, λ_{PN}	145321 (27830)	121761 - 168961
Annual incidence of illicit opioid use without OUD (but not from Rx-only opioid misuse) at baseline, λ_{IN}: baseline	10510 (3269)	8054 - 13188

© 2023 Chhatwal J et al. *JAMA Network Open.*

Annual percentage change in incidence of illicit opioid use without OUD (but not from Rx-only opioid misuse), λ_{IN}: apc	0.708 (0.18)	0.632 - 0.853
Annual transition rate from compartments *PN* to *IN*, p_{PN_IN}	0.00322 (0.00213)	0.000527 - 0.005
Annual transition rate from compartments *PN* to *OUD*, p_{PN_OUD}	0.0954 (0.041)	0.0681 - 0.129
Annual transition rate from compartments *IN* to *OUD*, p_{IN_OUD}	0.0858 (0.0761)	0.0222 - 0.124
Annual transition rate from compartments *OUD* to *R1*, p_{OUD_R1}	0.0709 (0.033)	0.0501 - 0.095
Annual exit rate from compartment *PN*, μ_{PN}	0.115 (0.0678)	0.0642 - 0.158
Annual exit rate from compartment *IN*, μ_{IN}	0.0973 (0.0851)	0.0273 - 0.143
Annual opioid overdose mortality rate from compartment *PN*, m_{PN}	0.000351 (0.000217)	0.000175 - 0.000515
Annual opioid overdose mortality rate from compartment *IN*, m_{IN}	0.0029 (0.00426)	0 - 0.00792
Annual opioid overdose mortality rate from compartment *OUD* (attributed to Rx-only opioids), m_{PD}	6.59e-05 (0.000164)	0 - 0
Annual opioid overdose mortality rate from compartment *OUD* (attributed to illicit opioids) at baseline, m_{ID}: baseline	0.00391 (0.000876)	0.00324 - 0.00467
Annual percentage change in opioid overdose mortality rate from compartment *OUD* (attributed to illicit opioids), m_{ID}: apc	0.424 (0.118)	0.308 - 0.538
Proportion of overdose deaths from recovery that are attributed to Rx-only opioids, q_{R_odRx}	0.335 (0.301)	0.0852 - 0.543
Scalar for opioid overdose mortality rate while on treatment, s_{odMOUD}	0.767 (0.661)	0.168 - 1.4

© 2023 Chhatwal J et al. *JAMA Network Open.*

eFigure 4. Simulation Band Created by the Mid-95th Percentile of the 1000 Calibrated Parameter Sets for Each State

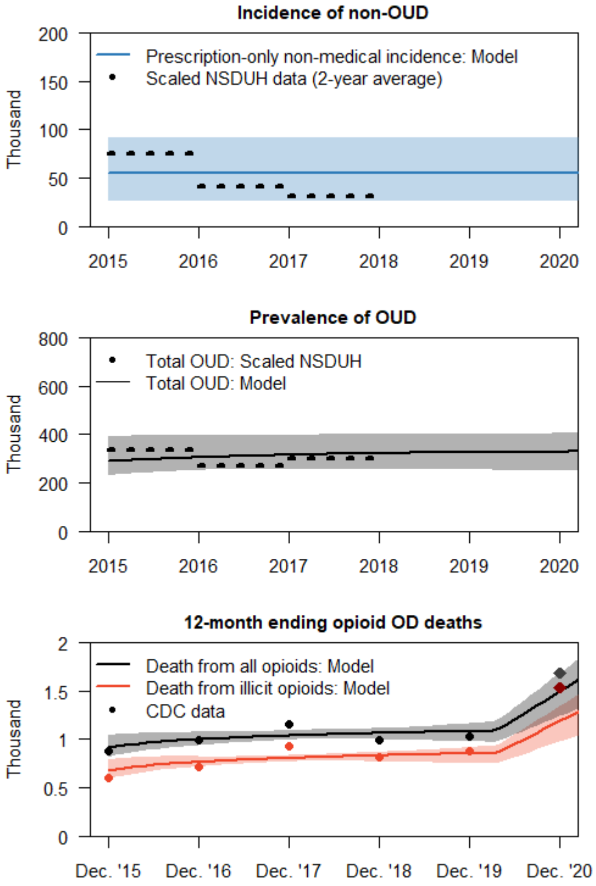

eFigure 4a. "Simulation band" created by the mid 95th percentile of the 1,000 calibrated parameter sets for Kentucky, obtained via Simulated Annealing searches.

© 2023 Chhatwal J et al. *JAMA Network Open.*

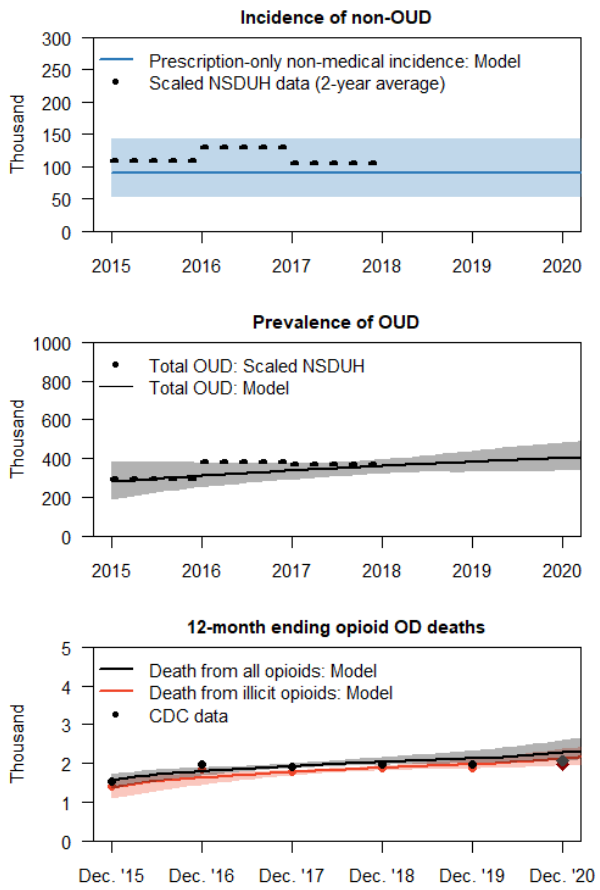

eFigure 4b. "Simulation band" created by the mid 95th percentile of the 1,000 calibrated parameter sets for Massachusetts, obtained via Simulated Annealing searches.

© 2023 Chhatwal J et al. *JAMA Network Open.*

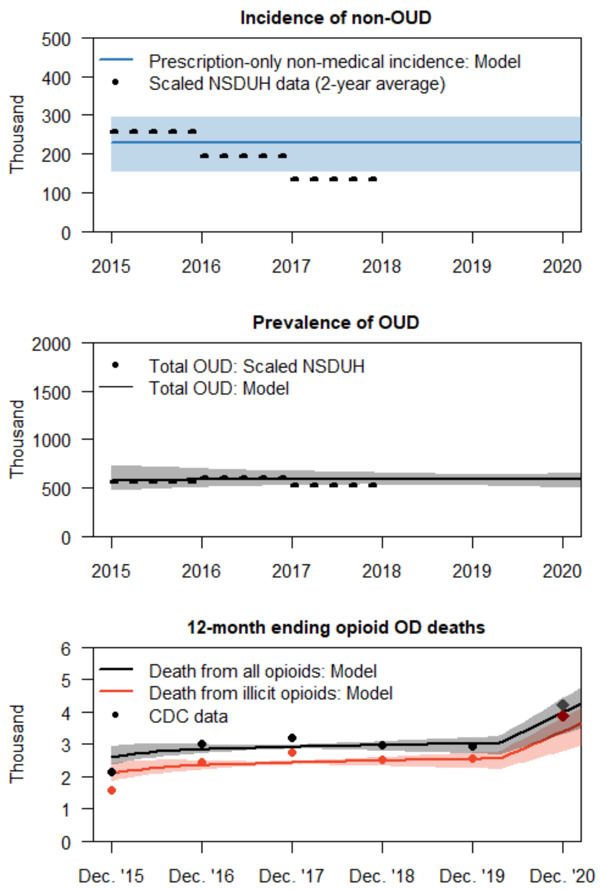

eFigure 4c. "Simulation band" created by the mid 95th percentile of the 1,000 calibrated parameter sets for New York, obtained via Simulated Annealing searches.

© 2023 Chhatwal J et al. *JAMA Network Open.*

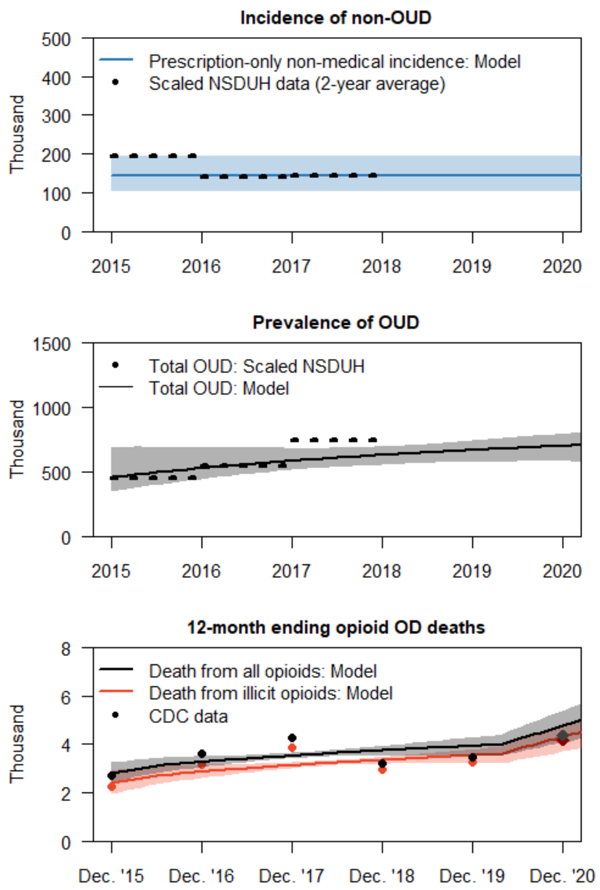

eFigure 4d. "Simulation band" created by the mid 95th percentile of the 1,000 calibrated parameter sets for Ohio, obtained via Simulated Annealing searches.

© 2023 Chhatwal J et al. *JAMA Network Open.*

eAppendix 3. Parameters for Model Projection

S3.1 Parameters for Projection Scenarios

Based on data and expert opinions outlined in the previous section, our transition rates were constant after 2017 and thus historic data did not suggest that our transition rates change during the model projections from 2021-2025. However, other events do cause some of our transition rates to change beginning in 2020. In section **S2.1** we detailed the impacts that COVID-19 had on opioid overdose mortality rates and MOUD initiation rates. Lastly, in the next section we detail the adjustments made to transition rates as a direct result of simulated interventions.

S3.2 Simulated Interventions to Reduce Opioid Overdose Deaths

One major priority of the HEALing Communities Study is to reduce the number of opioid overdose deaths through a multitude of interventions. In our study, we chose to focus on the following four interventions: **(1)** increasing the initiation rate of medications for opioid use disorder (MOUDs); **(2)** increasing the retention rate of MOUDs; **(3)** reducing overdose mortality rates through increased naloxone availability/usage; and **(4)** reducing the incidence of Rx opioid misuse. Prior to delving deeper in the details of each intervention, we next elaborate on the timings of interventions and the ramp-up of certain interventions to emulate the real-world steps to implement these interventions.

The HEALing Communities Study (HCS) timeline was impacted by the COVID-19 pandemic. Aligning the study's new timeline, we assumed that the first impact of the interventions occurred on August 1st, 2020, gradually ramped-up to full effectiveness by January 1st, 2021, and remained at full effectiveness until June 30th, 2022, or longer depending on which sustainability analysis we were performing (**eFigure 5**). Beyond which point, the effectiveness immediately dropped to zero by the next month (model cycle time step). This immediate drop-off is an assumption we made as this detail was not significant to our analyses but should be noted that this is not 100% realistic as interventions are likely to have some residual impact even when no longer being sustained.

During the ramp-up period, we assumed the effectiveness of any selected intervention obeyed the following smooth function:

$$f(x) = 6x^5 - 15x^4 + 10x^3, \quad 0 \le x \le 1,$$

where $x = 0$ corresponds to August 1st, 2020, $x = 1$ corresponds to January 1st, 2021, and which satisfies the following conditions:

Functional property	Explanation
• $f(0) = 0$:	• No impact of intervention on August 1st, 2020.
• $f(1) = 1$:	• Full effectiveness by January 1st, 2021.
• $f'(0) = f'(1) = 0$:	• Smooth start and finish to ramp-up.
• $g(x) := f\left(x + \frac{1}{2}\right) - \frac{1}{2}$ is an odd function:	• Rotationally symmetric around the midpoint where the effectiveness is at 50%.

One of the original goals of the HCS was to reduce overdose deaths by 40%. This lofty goal was set to occur over the calendar year of 2021 but was pushed back to cover the measurement period (or comparison window) of July 1st, 2021, through June 30th, 2022, because of delays from COVID-19. This updated measurement period is one we focus on in our analyses, but we also explore the impacts of sustaining interventions beyond June of 2022.

© 2023 Chhatwal J et al. *JAMA Network Open.*

eFigure 5. Timeline Used in the Opioid Policy Model (OPyM)

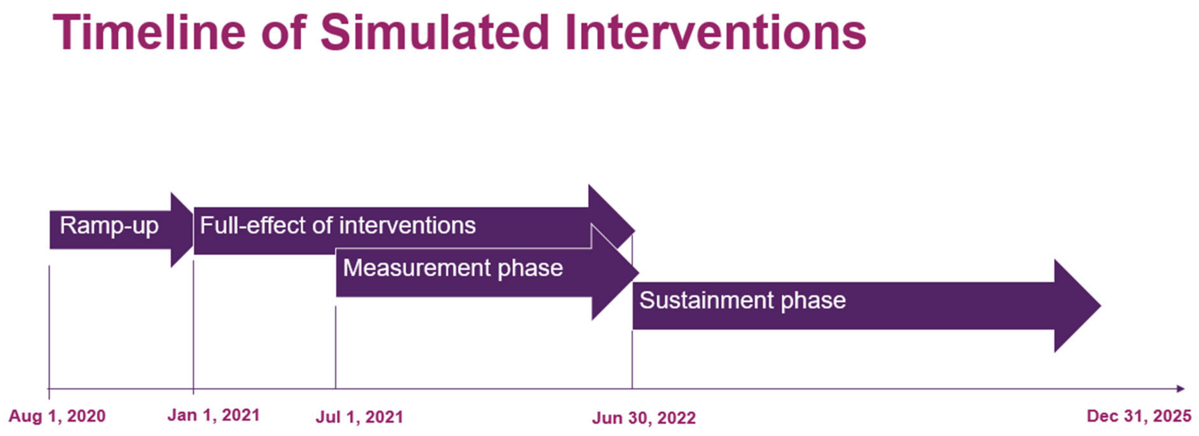

A smoothing function was applied to the ramp-up period to emulate the real-world process of introducing a new intervention.

Scaling up MOUD initiation rates

Increasing the initiation rate of MOUDs was done by applying a multiplication factor to the transition rate from compartments *OUD* to *R1* and *OUDrel* to *R1*. We assumed identical initiation rates for people with an opioid use disorder whether or not they just relapsed. Most of our analyses focused on scaling up both buprenorphine and methadone equally, in this case a 2-fold increase in MOUD initiation rates was a simple as multiplying the two previously mentioned transition rates by 2.

Preliminary findings of the HEALing Communities Study suggest that a 2-fold increase in MOUD initiations is the most realistic upper bound. To supplement our primarily analyses, we also explored a theoretical scenario where a 5-fold increase could be achieved (**eFigure 6**).

© 2023 Chhatwal J et al. *JAMA Network Open.*

eFigure 6. Temporal Trends in Opioid Overdose Deaths Under Status Quo and Implementation of Interventions, With and Without Sustainment for Different Durations

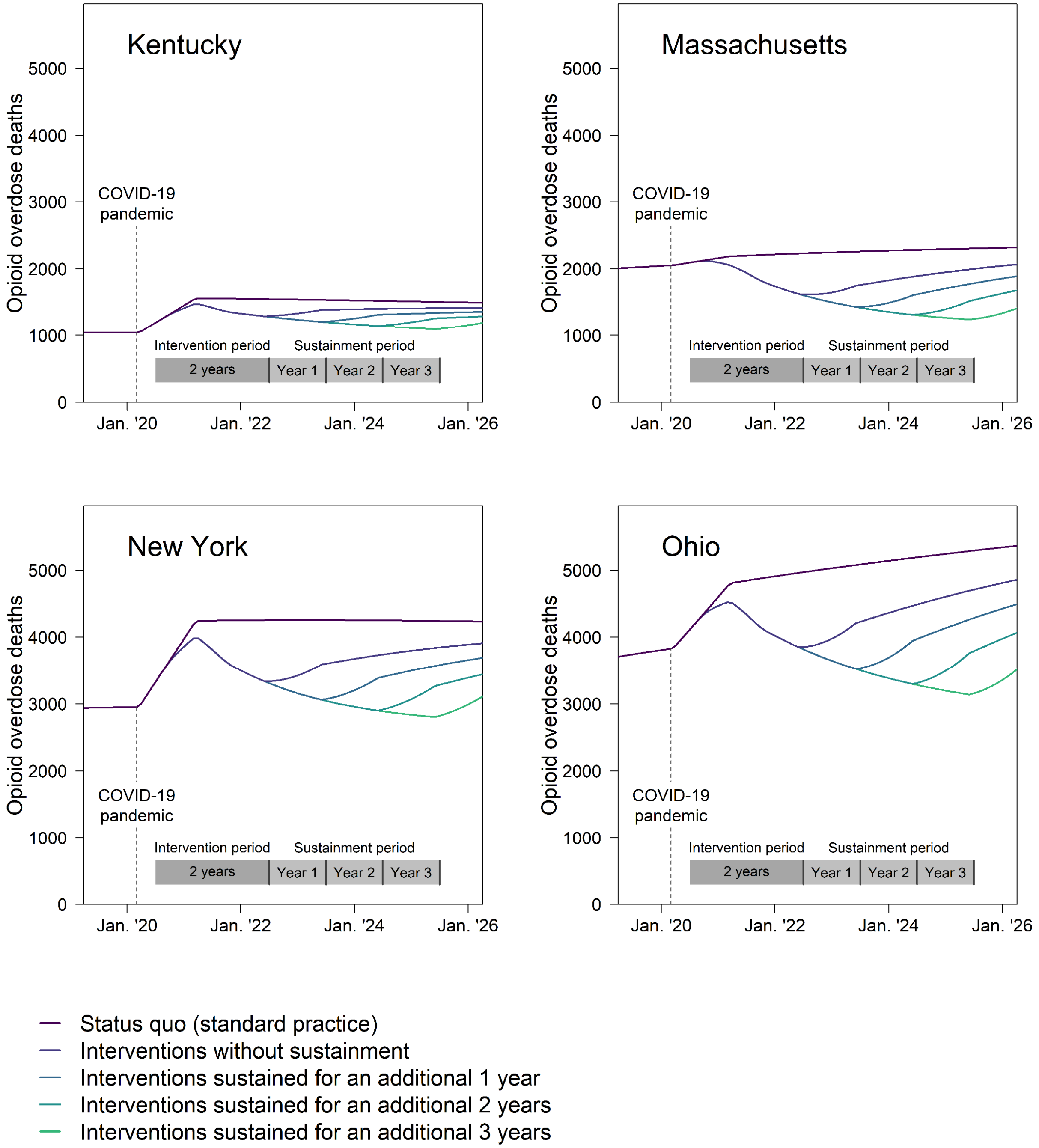

The selected intervention consists of 5 times increase in MOUD admissions, MOUD retention at the level observed in RCTs (6-month retention of 46% for buprenorphine and 74% for methadone), distribution of naloxone kits that translate to 10% mortality rate reduction, and 50% reduction in new prescription opioid misuse.

Scaling up MOUD retention rates

We considered two scenarios for MOUD retention rates: baseline retention rates derived in section **S2.**1 (6-month retention rates of 32% for BUP and 52% for MTD) and retention rates seen in randomized control trials (RCTs) (6-

© 2023 Chhatwal J et al. *JAMA Network Open.*

month retention rates of 46% for BUP and 74% for MTD). To scale up to retention rates found in RCTs, we took the month-by-month retention rates derived from Figure 1 of Morgan2018 and scaled them up together such that they used the same functional form but whose product of the first 6 retention rates by month was 46% for BUP and 74% for MTD.

Scaling up naloxone distribution

The efforts of our model were focused on an accurate simulation of OUD treatment and relapse, and as a trade-off it lacked a more thorough implementation of naloxone and non-fatal overdoses. Thus, we simply applied a multiplication scalar of 0.90 to all overdose mortality rates for intervention combinations involving naloxone. This scalar represents whatever combination of increased naloxone distribution and/or usage such that 10% of overdose deaths are prevented. The value of 10% was chosen from looking at studies of naloxone's impacts and reducing slightly as most of the HCS communities had significant naloxone availability prior to the HCS interventions.

Scaling up Rx opioid misuse prevention

In a previous version of our model, the impact of reducing the incidence of Rx opioid misuse was the main focus and was more thoroughly explored [2]. Due to the minimal impact of reducing the incidence of Rx opioid misuse seen in the previous study, we tested a significantly strong scenario where 50% of new Rx opioid misuse was prevented through interventions.

© 2023 Chhatwal J et al. *JAMA Network Open.*

eReferences

1. Homer JB, Hirsch GB. System Dynamics Modeling for Public Health: Background and Opportunities. *American Journal of Public Health*. 2006;96(3):452-458. doi:10.2105/ajph.2005.062059
2. Chen Q, Larochelle MR, Weaver DT, et al. Prevention of Prescription Opioid Misuse and Projected Overdose Deaths in the United States. *JAMA Netw Open*. Feb 1 2019;2(2):e187621. doi:10.1001/jamanetworkopen.2018.7621
3. U.S. Department of Health and Human Services, Substance Abuse and Mental Health Services Administration, Center for Behavioral Health Statistics and Quality. National Survey on Drug Use and Health, Personal Communication. Accessed 2020.
4. U.S. Department of Health and Human Services, Substance Abuse and Mental Health Services Administration, Center for Behavioral Health Statistics and Quality. National Survey on Drug Use and Health: Model-Based Prevalence Estimates (50 States and the District of Columbia). Accessed July 26, 2022. https://www.samhsa.gov/data/nsduh/state-reports-NSDUH-2020
5. Centers for Disease Control and Prevention, National Center for Health Statistics. Multiple Cause of Death 1999-2019 on CDC WONDER online database. Accessed July 26, 2022. https://wonder.cdc.gov/mcd-icd10.html
6. U.S. Department of Justice, Drug Enforcement Administration. Automated Reports and Consolidated Ordering System (ARCOS) Retail Drug Summary Reports. Accessed July 26, 2022. https://www.deadiversion.usdoj.gov/arcos/retail_drug_summary/index.html
7. U.S. Department of Health and Human Services, Substance Abuse and Mental Health Services Administration. National Survey of Substance Abuse Treatment Services (N-SSATS). Accessed July 26, 2022. https://www.datafiles.samhsa.gov/
8. Timko C, Schultz NR, Cucciare MA, Vittorio L, Garrison-Diehn C. Retention in medication-assisted treatment for opiate dependence: A systematic review. *J Addict Dis*. 2016;35(1):22-35. doi:10.1080/10550887.2016.1100960
9. Morgan JR, Schackman BR, Leff JA, Linas BP, Walley AY. Injectable naltrexone, oral naltrexone, and buprenorphine utilization and discontinuation among individuals treated for opioid use disorder in a United States commercially insured population. *J Subst Abuse Treat*. Feb 2018;85:90-96. doi:10.1016/j.jsat.2017.07.001
10. Ronquest NA, Willson TM, Montejano LB, Nadipelli VR, Wollschlaeger BA. Relationship between buprenorphine adherence and relapse, health care utilization and costs in privately and publicly insured patients with opioid use disorder. *Subst Abuse Rehabil*. 2018;9:59-78. doi:10.2147/sar.S150253
11. Sordo L, Barrio G, Bravo MJ, et al. Mortality risk during and after opioid substitution treatment: systematic review and meta-analysis of cohort studies. *Bmj*. Apr 26 2017;357:j1550. doi:10.1136/bmj.j1550
12. Barocas JA, White LF, Wang J, et al. Estimated Prevalence of Opioid Use Disorder in Massachusetts, 2011-2015: A Capture-Recapture Analysis. *Am J Public Health*. Dec 2018;108(12):1675-1681. doi:10.2105/ajph.2018.304673
13. Keyes KM, Rutherford C, Hamilton A, et al. What is the prevalence of and trend in opioid use disorder in the United States from 2010 to 2019? Using multiplier approaches to estimate prevalence for an unknown population size. *Drug Alcohol Depend Rep*. Jun 2022;3doi:10.1016/j.dadr.2022.100052
14. Julien J, Ayer T, Tapper EB, Barbosa C, Dowd WN, Chhatwal J. Effect of increased alcohol consumption during COVID-19 pandemic on alcohol-associated liver disease: A modeling study. *Hepatology*. December 8, 2021 2021;75:1480-1490. doi: https://doi.org/10.1002/hep.32272
15. Currie JM, Schnell MK, Schwandt H, Zhang J. Prescribing of Opioid Analgesics and Buprenorphine for Opioid Use Disorder During the COVID-19 Pandemic. *JAMA Network Open*. 2021;4(4):e216147-e216147. doi:10.1001/jamanetworkopen.2021.6147
16. Mark TL, Gibbons B, Barnosky A, Padwa H, Joshi V. Changes in Admissions to Specialty Addiction Treatment Facilities in California During the COVID-19 Pandemic. *JAMA Network Open*. 2021;4(7):e2117029-e2117029. doi:10.1001/jamanetworkopen.2021.17029

© 2023 Chhatwal J et al. *JAMA Network Open.*
