# Supplementary material for: Estimated Reductions in Opioid Overdose Deaths With Sustainment of Public Health Interventions in 4 US States
Source: JAMA Netw Open. 2023 Jun 9;6(6):e2314925. doi: 10.1001/jamanetworkopen.2023.14925 (PMC10257094; doi:10.1001/jamanetworkopen.2023.14925)
Supplement: Supplement 2. — Data Sharing Statement [file jamanetwopen-e2314925-s002.pdf]

Data Sharing Statement

Chhatwal. Estimated Reductions in Opioid Overdose Deaths With Sustainment of Public Health Interventions in 4 US States. *JAMA Netw Open*. Published June 09, 2023. doi:10.1001/jamanetworkopen.2023.14925

Data
Data available: Yes
Data types: Data (not involving human participants)
How to access data: All data are made available in the manuscript
When available: With publication

Supporting Documents
Document types: None

Additional Information
Who can access the data: researchers whose proposed use of the data has been approved
Types of analyses: for research purpose
Mechanisms of data availability: All data are made available in the manuscript
